# Old mitochondria regulate niche renewal via α-ketoglutarate metabolism in stem cells

**DOI:** 10.1038/s42255-025-01325-7

**Published:** 2025-07-14

**Authors:** Simon Andersson, Hien Bui, Arto Viitanen, Daniel Borshagovski, Ella Salminen, Sami Kilpinen, Angelika Gebhart, Emilia Kuuluvainen, Swetha Gopalakrishnan, Nina Peltokangas, Martyn James, Kaia Achim, Eija Jokitalo, Petri Auvinen, Ville Hietakangas, Pekka Katajisto

**Affiliations:** 1 Faculty of Biological and Environmental Sciences, University of Helsinki, Helsinki, Finland; 2 Institute of Biotechnology, HiLIFE, University of Helsinki, Helsinki, Finland; 3 Department of Cell and Molecular Biology, Karolinska Institutet, Stockholm, Sweden

**Keywords:** Intestinal stem cells, Stem-cell niche, Metabolism, Mitochondria

## Abstract

Cellular metabolism is a key regulator of cell fate^1^, raising the possibility that the recently discovered metabolic heterogeneity between newly synthesized and chronologically old organelles may affect stem cell fate in tissues^2,3^. In the small intestine, intestinal stem cells (ISCs)^4^ produce metabolically distinct progeny^5^, including their Paneth cell (PC) niche^6^. Here we show that asymmetric cell division of mouse ISCs generates a subset enriched for old mitochondria (ISC^mito-O^), which are metabolically distinct, and form organoids independently of niche because of their ability to recreate the PC niche. ISC^mito-O^ mitochondria produce more α-ketoglutarate, driving ten-eleven translocation-mediated epigenetic changes that promote PC formation. In vivo α-ketoglutarate supplementation enhanced PC turnover and niche renewal, aiding recovery from chemotherapy-induced damage in aged mice. Our results reveal a subpopulation of ISCs whose old mitochondria metabolically regulate cell fate, and provide proof of principle for metabolically promoted replacement of specific aged cell types in vivo.

## Abstract (web summary)

Andersson et al. show that intestinal stem cells enriched for old mitochondria are metabolically distinct and have enhanced ability to regenerate the epithelial niche.

## Main

Stem cells and their differentiated progeny differ metabolically; recent studies indicate that metabolic rewiring controls changes in cell fate (reviewed in ref. ^7^). However, whether the functional heterogeneity found in tissue stem cells is regulated by metabolism is unclear. We recently found that in cultured mammary epithelial cells, mitochondria of distinct chronological age classes^2^ are metabolically different^3^ and affect cell fate decisions. This raises the question whether mitochondrial age also influences metabolism and cell fate choices in tissue stem cells. Interestingly, intestinal stem cells (ISCs) harbour functional heterogeneity^8,9^, but the metabolic traits separating actively and slowly dividing ISCs are only beginning to be understood^10^; the role of metabolism as a cell fate determinant in actively dividing ISCs remains unsettled^11,12^. In this study, we found that the age composition of mitochondria creates metabolic and functional diversity among ISCs. Asymmetric ISC divisions enrich old mitochondria into a subset of daughter cells, which promotes their ability to regenerate the epithelial niche. Consequently, asymmetrical segregation of distinct mitochondrial age classes forms a facet of tissue stem cell regulation and, as we show, opens opportunities to metabolically guide the replacement of defective cell types in tissues.

**Fig. 1:**
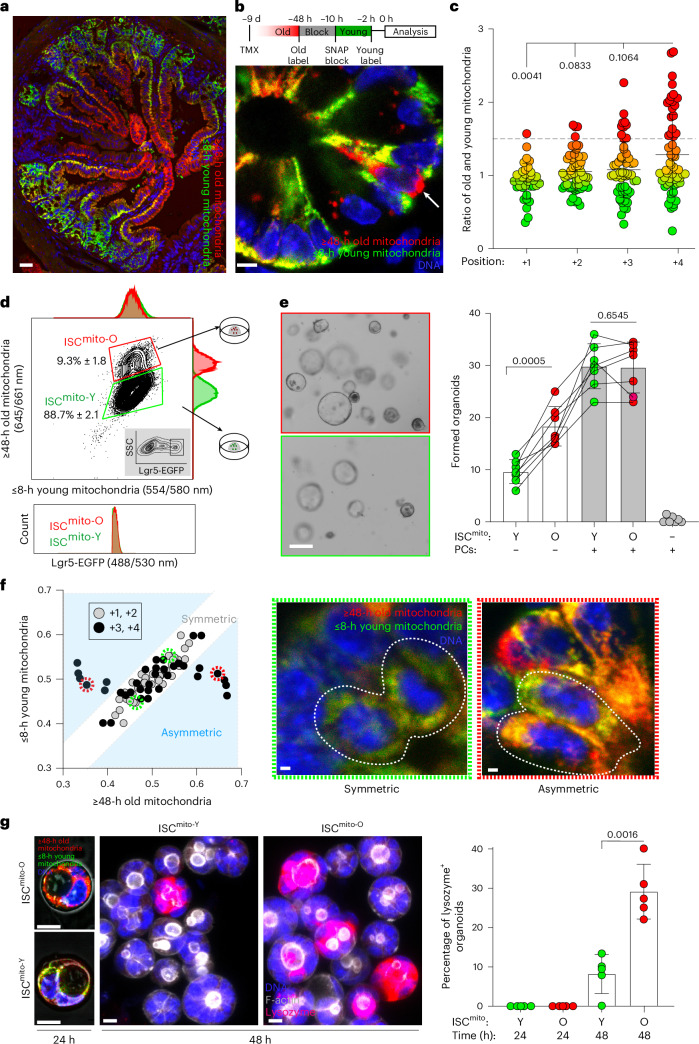
Old mitochondria induce the niche independence of stem cells. **a**, Tissue section of the mitochondrial age gradient in the small intestine, repeated independently three times (blue, 4′,6-diamidino-2-phenylindole (DAPI); red, ≥48-h old mitochondria; green, ≤8-h young mitochondria). **b**, Top, Schematic of in vivo labelling of mitochondrial age classes. Bottom, Subset of ISCs highly enriched for old mitochondria (arrow) in tissue labelled in vivo (blue, DAPI; red, ≥48-h old mitochondria; green, ≤8-h young mitochondria). **c**, Ratio of old (≥48 h) and young (≤8 h) mitochondrial ISCs at four positions, starting from the crypt bottom and centre towards the niche border (positions 1–4) in tissue labelled in vivo. A one-way analysis of variance (ANOVA) with two-tailed Dunnett’s post-hoc test was used to compare to position 4. Data are shown as the mean ± s.d. (*n* = 5, all data points shown; minimum 40 cells per mouse). **d**, Fluorescence-activated cell sorting (FACS) analysis of Lgr5-EGFP^hi^ ISCs (2% contour plot) (inset: parent gate, 5% contour plot) indicated a distinct ISC population enriched for old mitochondria (ISC^mito-O^). Data are shown as the mean ± s.d. ISC^mito-O^ frequency of Lgr5-EGFP^hi^ (the same as in Extended Data Fig. 1h) is shown. Histograms: young (top) and old (side) mitochondria and Lgr5-EGFP (bottom) from ISC^mito-O^ (red) and ISC^mito-Y^ (green). **e**, Niche-dependent and niche-independent regenerative growth of single ISCs at day 6 (ISC^mito-O^ (O) and ISC^mito-Y^ (Y)). Representative images on day 6. A two-tailed, paired Student’s *t*-test (*n* = 7) without correction for multiple comparisons was used. Data are the absolute number of organoids and shown as the mean ± s.d. **f**, Age-selective apportioning of mitochondria (blue background) occurred at the +3/+4 crypt position. Left: each division pair is shown as the relative amount inherited out of the total. Middle, Representative image of symmetric apportioning. Right: asymmetric apportioning. Data are from three independent experiments. **g**, PC emergence at 48-h ISC^mito-O^ (O) and ISC^mito-Y^ (Y)-initiated organoids. Representative images from 24 h (left: blue, DAPI; red, ≥48-h old; green, ≤8-h young mitochondria; grey, transmitted light) and 48 h (middle right: blue, DAPI; magenta, lysozyme; white, phalloidin). A two-tailed, paired Student’s *t*-test (*n* = 5) was used. Data are shown as the mean ± s.d. Scale bars, 50 µm (**a**), 5 µm (**b**,**g**), 20 μm (**e**), 1 µm (**f**). Source data

**Extended Data Fig. 1:**
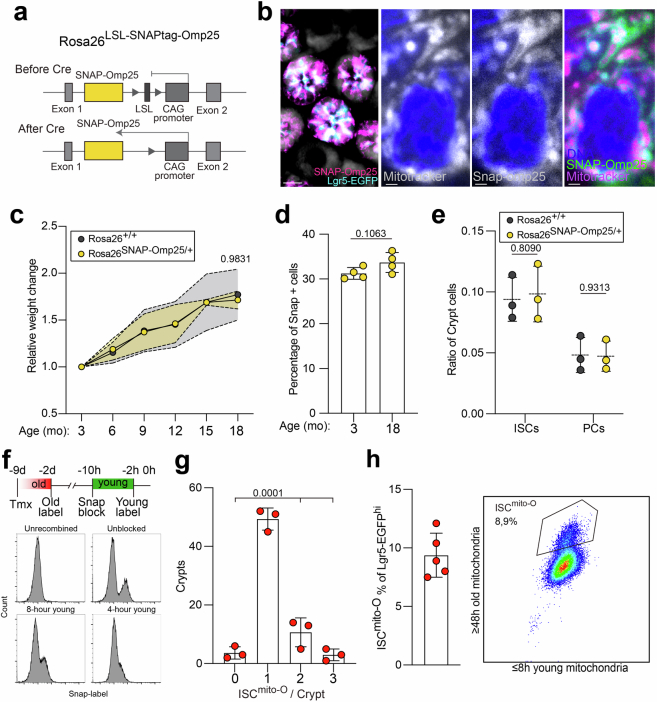
Characterization of SNAP-Omp25 mouse. **a**, Schematic of lox-Stop-lox-SNAPtag-Omp25-construct. **b**, Left, Representative image of SNAP-Omp25 expression in Lgr5-EGFP positive crypts 5 days after a single dose of 80 mg/kg of tamoxifen in a Lgr5^EGFP-IRES-creERT2/+^Rosa26^lox-Stop-lox-SNAPtag-omp25/+^ mouse repeated independently four times. Scale bar 25 µm. Right, Representative image of SNAP-Omp25 localization to the outer membrane of mitochondria in organoids. Repeated independently three times. Scale bar 1 µm. **c**, Relative weight change over 18 months after a single dose of 80 mg/kg of tamoxifen. Monthly data points represent mean and colored area between dashed lines show the 50% interquartile range). (*n* = 6) two-tailed Student’s unpaired *t*-test at 18 months **d**, Flow cytometer frequencies of SNAP+ cells out of LiveEpcam+ cells two weeks after recombination (3 months) and 15 months after recombination (18 months) (*n* = 4) two-tailed Student’s unpaired *t*-test. Data mean ± SD. **e**, Flow cytometer frequencies of crypt cells: Paneth cells (CD24hiSSChi) and ISCs (Cd24medLgr5-EGFPhi) out of LiveEpcam+ cells. (*n* = 3) Data mean±S.D. **f**, Top, Schematic of *in vivo* labelling for mitochondrial age classes. Bottom, different labelling time-windows to establish optimal young mitochondria label (histogram showing Snap staining when young label is given at indicated time after snap-block.) **g**, Frequencies of crypts with indicated number of ISCs^mito-O^ in *in vivo* labelled tissue. (*n* = 3) One-way ANOVA with two-tailed Dunnett’s post-hoc test. Data mean±S.D. **h**, Left, Flow cytometer frequencies of ISCs^mito-O^ out of all ISCs (Cd24medLgr5-EGFPhi). (*n* = 5). Data mean ± SD. Right, Representative FACS pseudo color plot of ISCs^mito-O^ gating out of all ISCs (Cd24medLgr5-EGFPhi). For full sort scheme see Supplementary Fig. 1. Source data

To study whether metabolically distinct age classes of mitochondria are found in vivo, we generated a knock-in mouse model where a *lox*-Stop-*lox*-SNAPtag-Omp25 construct is introduced to the *Rosa26* locus (Extended Data Fig. 1a). We crossed these mice with *Lgr5*-EGFP-IRES-creERT2 reporter mice, which allows the identification of *Lgr5*-EGFP^hi^ ISCs^4^ and tamoxifen (TMX)-induced expression of SNAPtag in stem cells and their progeny (Extended Data Fig. 1b). SNAPtag-Omp25 localizes to the mitochondrial outer membrane and allows for sequential temporal labelling of mitochondria^2^ by cell-permeable fluorescent SNAPtag substrates^13^ (Extended Data Fig. 1b). Mice developed normally and SNAPtag expression was stable for 18 months without impact on mouse well-being or intestinal cell composition (Extended Data Fig. 1c–e). To distinguish mitochondrial age classes, we injected mice with two SNAP substrates and an intermediate blocking reagent intraperitoneally, and designated mitochondria with their retained SNAP label after 48 h as old, and mitochondria labelled in the shortest technically feasible window of 8 h as young (Extended Data Fig. 1f). Reflecting the rapid renewal of the absorptive epithelium, mitochondrial age composition formed a gradient along the crypt–villus axis, with cells in villi containing predominantly old mitochondria and proliferative crypts having younger mitochondria (Fig. 1a). However, while most ISCs at the bottom of the crypt contained young mitochondria, a subset of ISCs located in the upper part of the stem cell niche was surprisingly enriched for old mitochondria (Fig. 1b,c). We designated these cells ISC^mito-O^ and found that most crypts contained 1–2 such cells (Extended Data Fig. 1g). In accordance, flow cytometry analysis of Lgr5^hi^ ISCs revealed that the distinct ISC^mito-O^ subpopulation represents approximately 9% of all ISCs (Fig. 1d and Extended Data Fig. 1h).

**Extended Data Fig. 2:**
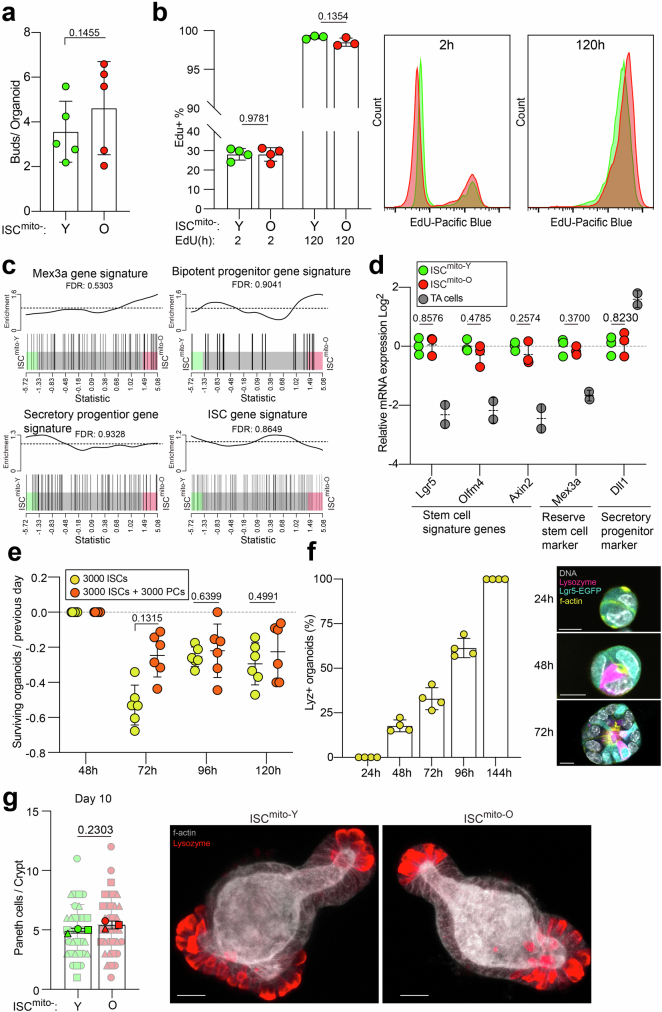
Reserve stem cell profiling and Paneth cell emergence dynamics. **a**, Organoid budding at 10 days of culture is not affected by mitochondrial age composition of initiating ISC. Two-tailed Student’s paired *t*-test (*n* = 5). Data mean±S.D. **b**, Left, Flow cytometer frequencies of EdU+ cells in ISCs^mito-O^ and ISCs^mito-Y^ populations after one 10 mg/kg dose of EdU (2 h, *n* = 4), or continuous labelling of 0.2 mg/ml EdU via drinking water over five days (120 h, *n* = 3). Two-tailed Student’s paired *t*-test. Data mean±S.D. Right, representative histograms of EdU labelled cells in ISCs^mito-O^ and ISCs^mito-Y^ populations after 2 h or 120 h of labelling. **c**, GSEA preranked analysis of ISC^mito-O^ versus ISCs^mito-Y^ for the gene lists ‘Mex3a gene signature’, ‘Bipotent progenitor gene signature’, ‘Label retaining secretory progenitor’, ‘Stem cell signature’. See methods for source of gene lists. False discovery rate (FDR) is shown (*n* = 6) **d**, Relative expression of stem-cell signature, reserve stem cell, and secretory progenitor marker genes in isolated ISCs^mito-O^ and ISCs^mito-Y^ and EGFPLo transit amplifying (TA) cells. (*n* = 3). Two-tailed Student’s paired *t*-test. Log_2_ normalized to ISCs^mito-Y^, data mean ± SD. **e**, Survival frequency of organoids compared to previous day initiated by isolated single ISCs or ISCs co-cultured with Paneth cells (*n* = 6). Two-tailed Student’s paired *t*-test. Data mean ± SD. **f**, Left, Paneth cells emerge at two days of culture and by six days all organoids contain Paneth cells. Data mean ± SD, n = 4 independent mice. Right, representative images of organoids at indicated times of culture initiated from sorted single ISCs (Grey=DAPI, magenta=lysozyme, cyan=EGFP, yellow=phalloidin). Scale bars 5 µm. **g**, Left, Paneth cell number is not different between ISCs^mito-O^ and ISCs^mito-Y^ initiated mature organoids at ten days of culture. Two-tailed Student’s paired *t*-test(*n* = 3). Comparing organoids initiated by ISC^mito-O^ (O) and ISC^mito-Y^ (Y) cells from three mice (indicated with symbols). Data mean±S.D. Data of individual organoids shown in opaque. Right, representative images organoids at ten days of culture (red lysozyme, white phalloidin). Scale bar 25 µm. Source data

To assess the impact of mitochondrial age composition, we first used the capacity of isolated single ISCs to form clonogenic organoids^6^ as an in vitro proxy of intrinsic ISC capacity (Fig. 1d). Strikingly, the organoid-forming capacity of ISC^mito-O^ was higher than that of stem cells with young mitochondria (ISC^mito-Y^) (Fig. 1e). After formation, mature organoids begin to bud new crypt domains, providing a measure for the prolonged regenerative potential of the formed organoid. We noted no difference in budding of organoids from ISC^mito-O^ or ISC^mito-Y^ after 10 days of culture (Extended Data Fig. 2a), suggesting that mitochondrial age affects the initial stages of organoid formation. As ISC function is also controlled by neighbouring Paneth cells (PCs) that produce stem cell-regulating factors^6^, we next investigated the niche-coupled organoid-forming capacity of ISC^mito-O^ and ISC^mito-Y^ in co-cultures with PCs. Intriguingly, mitochondrial age composition provided no advantage in niche-coupled organoid formation (Fig. 1e), implying that enrichment of old mitochondria offers a distinct advantage to ISC^mito-O^ to form an organoid niche independently. Taken together, these data suggest that mitochondrial age composition acts transiently in organoid formation and affects niche-independent regeneration specifically.

Niche independence is a trait attributed to reserve stem cells that are less reliant on niche signals^14^ and can regenerate the epithelium after damage^9,14,15^. A slow cell cycle is a reported hallmark of such reserve stem cells in the intestine^9,14,15^, but we found that ISC^mito-O^ contained the same frequency of 5-ethynyl-2′-deoxyuridine (EdU)-labelled cells as the rest of the ISC population (Extended Data Fig. 2b). Moreover, the markers and expression profiles of reserve stem cells (*Mex3a*)^9^, early secretory progenitors (*Dll1*)^16^ and bipotent progenitors^17^, or the Wnt and Notch signalling pathway markers of stem cells (*Lgr5* and *Olfm4* respectively)^18^, did not distinguish ISC^mito-O^ from other ISCs (Extended Data Fig. 2c,d). Jointly, these data suggest that the age of mitochondria can be used to identify the heterogeneity and subdivision of ISCs that had previously gone unnoticed.

The similar EdU labelling (Extended Data Fig. 2b) indicated that ISC^mito-O^ and ISC^mito-Y^ have similar divisions rates, raising the question of how a subset of actively dividing cells becomes enriched with old mitochondria? To address this, we analysed mitochondrial apportioning during ISC divisions in vivo. Strikingly, while the old and young mitochondria of the mother cell were apportioned symmetrically in most ISC divisions, 15% of divisions resulted in daughter cells with asymmetric enrichment of young and old mitochondria (Fig. 1f). Moreover, we noted such asymmetric and age-selective apportioning of mitochondria only in divisions occurring at the +3 or +4 positions (Fig. 1f), which correlated with the location of ISC^mito-O^ (Fig. 1c).

ISCs have been predicted to divide symmetrically with each division, generating two equipotent daughter cells^19,20^. To probe the functional consequences of the age-selective segregation of mitochondria in some ISC divisions, we focused on the ability of ISC^mito-O^ to form organoids without PCs (Fig. 1e). To maintain or restore tissue homeostasis, stem cells ultimately require support from their niche^21^. Correspondingly, ISCs that are challenged to regenerate the tissue without initial help from the niche, that is, niche independently, must eventually also regenerate their niche including PCs^22^. In organoid culture, where the mesenchymal niche is substituted by factors of the medium, we found the strongest bottleneck for organoid initiation (starting with just ISCs) to occur right after the time when the first PCs emerge (Extended Data Fig. 2e,f). Co-culturing ISCs with PCs overcame the bottleneck, suggesting that PC emergence is in fact a crucial step of niche-independent regeneration. Importantly, at this crucial time, ISC^mito-O^ had regenerated three times as many PCs containing organoids as ISC^mito-Y^ (Fig. 1g). However, after the first in vitro division at 24 h, no PC had emerged (Fig. 1g), indicating that ISCs^mito-O^ are not fully determined to the PC fate but biased for their emergence. Furthermore, the number of PCs in mature organoids after 10 days of culture was similar (Extended Data Fig. 2g), supporting the notion that mitochondrial age composition affects niche-independent organoid formation only transiently. Taken together, these data uncover mitochondrial age composition as a new aspect determining ISC function and suggests that old mitochondria promote niche independence by facilitating regeneration of lost niche cells.

**Fig. 2:**
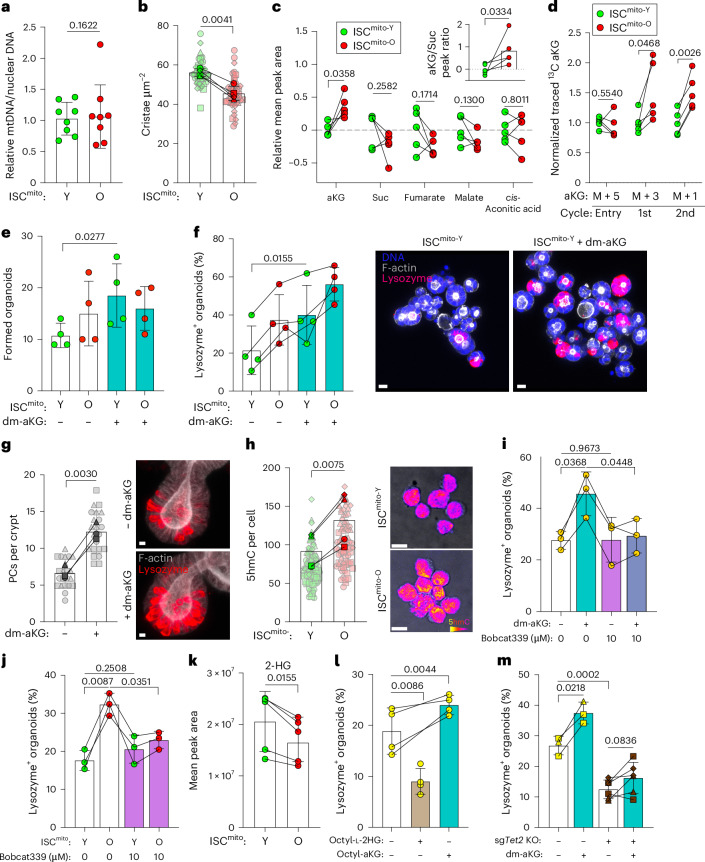
aKG in ISC^mito-O^ contributes to TET-mediated DNA hydroxymethylation in ISCs, promoting PC emergence. **a**, mtDNA in ISC^mito-O^(O) and ISC^mito-Y^ (Y) cells. A two-tailed, paired Student’s *t*-test (*n* = 8) was used. Data are shown as the mean ± s.d. **b**, Density of mitochondrial cristae using TEM. A two-tailed, paired Student’s *t*-test (*n* = 4) was used. ISC^mito-O^ (O) and ISC^mito-Y^ (Y) cells from four mice (symbol shape). Data are shown as the mean ± s.d.; individual cells are shown in opaque. **c**, Liquid chromatography–mass spectrometry (LC–MS) analysis of selected TCA cycle intermediates. log_2_ peak areas relative to ISCs^mito-Y^ are shown. Inset, aKG/Suc. A two-tailed, paired Student’s *t*-test (*n* = 5) was used. Data are shown as the mean ± s.d. **d**, LC–MS analysis of the TCA cycling rate in ISCs^mito-O^ and ISCs^mito-Y^. aKG at entry (^13^C_5_), after the first cycle (^13^C_3_) and after second cycle (^13^C_1_) was traced from ^13^C_5_-glutamine. Peak areas were level-normalized in ISC^mito-Y^. A two-tailed, paired Student’s *t*-test (*n* = 5) was used. Data are shown as the mean ± s.d. **e**, Niche-independent growth at day 6 of ISC^mito-O^ (O) and ISC^mito-Y^ (Y) supplemented with dm-aKG for 48 h. A two-tailed, paired Student’s *t*-test (*n* = 4) was used. Data are the absolute number of organoids shown as the mean ± s.d. **f**, PC emergence after 48 h of dm-aKG-supplemented, niche-independent growth of ISC^mito-O^ (O) and ISC^mito-Y^ (Y). A two-tailed, paired Student’s *t*-test (*n* = 4) was used. Data are shown as the mean ± s.d. Representative images at 48 h (blue, nuclei; magenta, lysozyme; white, phalloidin). **g**, PC quantification in organoids after 6 days of dm-aKG treatment. Quantification and representative images at 6 days (red, lysozyme; white, phalloidin). A two-tailed, paired Student’s *t*-test was used (*n* = 3 mice (symbol shape) with individual crypts (opaque)). Data are shown as the mean ± s.d. **h**, Nuclear 5hmC in ISCs^mito-O^ (O) and ISC^mito-Y^ (Y). Representative images of cells (yellow, high; purple, low). A two-tailed, paired Student’s *t*-test (*n* = 4) was used to compare ISC^mito-O^ (O) and ISC^mito-Y^ (Y) cells from four mice (symbol shape). Data are shown as the mean ± s.d.; individual cells are shown in opaque. **i**, PC emergence in niche-independent culture of Lgr5-EGFP^hi^ ISCs supplemented with dm-aKG and Bobcat339 for 48 h. A two-tailed, paired Student’s *t*-test (*n* = 3) was used. Data are shown as the mean ± s.d. **j**, PC emergence from ISCs^mito-O^ (O) and ISCs^mito-Y^ (Y) supplemented with Bobcat339 for 48 h. A two-tailed, paired Student’s *t*-test (*n* = 3) was used. Data are shown as the mean ± s.d. **k**, ISC^mito-O^ had decreased 2HG (LC–MS mean peak area; *n* = 5). A two-tailed, paired Student’s *t*-test was used. Data are shown as the mean ± s.d. **l**, PC emergence in niche-independent culture of ISCs supplemented with Octyl-l-2HG or Octyl-aKG for 48 h. A two-tailed, paired Student’s *t*-test (*n* = 4) was used. Data are shown as the mean ± s.d. **m**, PC emergence of lentivirally targeted CRISPR–Cas9 *Tet2* knockout (KO) or Scramble (Scr) CD24^med^SSC^med^-sorted cells ± dm-aKG. A two-tailed, paired Student’s *t*-test (Scr, *n* = *3*; sgTET2 KO, *n* = 6) was used. Data are shown as the mean ± s.d. from three independent transductions (represented by the shapes and repeated twice per mouse). Scale bar, 5 μm (**f**,**g**,**h**). Source data

**Extended Data Fig. 3:**
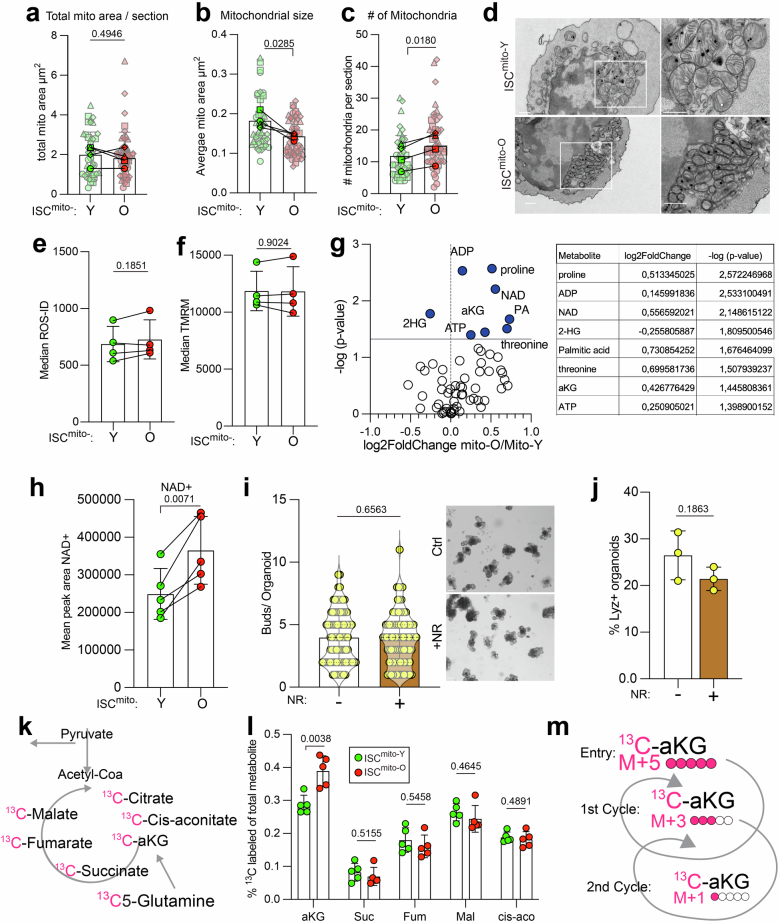
Mitochondrial and metabolic comparisons. **a**, TEM quantification of total mitochondrial profile area per cell section in sorted ISC^mito-O^ (O) and ISC^mito-Y^ (Y). Two-tailed Student’s paired *t*-test (*n* = 4). Comparing ISC^mito-O^ (O) and ISC^mito-Y^ (Y) cells from four mice (indicated with symbols). Data mean±S.D. individual cells shown in opaque. **b**, TEM quantification of mean mitochondrial profile area per cell section. Two-tailed Student’s paired *t*-test (*n* = 4). Comparing ISC^mito-O^ (O) and ISC^mito-Y^ (Y) cells from four mice (indicated with symbols). Data mean±S.D. individual cells shown in opaque. **c**, TEM quantification of total number of mitochondria profiles per cell section. Two-tailed Student’s paired *t*-test (*n* = 4). Comparing ISC^mito-O^ (O) and ISC^mito-Y^ (Y) cells from four mice (indicated with symbols). Data mean±S.D. individual cells shown in opaque. **d**, Representative TEM images of ISCs^mito-O^ and ISCs^mito-Y^. Scale bars 0,5 µm. **e**, Flow cytometer analysis of superoxide (ROS-ID) in ISCs^mito-O^ and ISCs^mito-Y^. Two-tailed Student’s paired *t*-test (*n* = 4). Data median±SD. **f**, Flow cytometer analysis of mitochondrial membrane potential (TMRM) in ISCs^mito-O^ and ISCs^mito-Y^. Two-tailed Student’s paired *t*-test (*n* = 4). Data median±SD. **g**, Metabolomics analysis of ISCs^mito-O^ and ISCs^mito-Y^ plotted as a volcano plot of -log(p-value((*n* = 5) two-tailed Student’s paired *t*-test) (Y-axis) and Log_2_ mean fold change (X-axis) (significance p-value < 0.05). **h**, ISC^mito-O^ have increased NAD+ (LC–MS mean peak area) (*n* = 5, two-tailed Student’s paired *t*-test. Data mean ± SD. **i**, Nicotinamide riboside (NR) supplementation for 6 days does not impact organoid regeneration (data from 3 biological repeats each data point is one organoid two-tailed Student’s *t*-test). Right, representative brightfield images of organoids at day 6, scale bar 50 µm. **j**, Paneth cells emergence after 48 h of NR supplemented niche-independent regenerative growth of sorted ISCs. Two-tailed Student’s paired *t*-test (*n* = 3). Data are mean ±S.D. **k**, Schematic of tracing from ^13^C_5_-Glutamine into TCA-cycle metabolites. **l**, TCA-metabolite labelling frequency out of total from ^13^C_5_-Glutamine. (*n* = 5) two-tailed Student’s paired *t*-test. Data mean ± SD. **m**, Schematic of tracing multiple rounds of TCA-cycle from ^13^C_5_-Glutamine. Source data

To better understand the mitochondrial regulation of niche-independent regeneration, we sought to investigate how mitochondrial morphology, content and metabolism differ between ISC^mito-O^ and ISC^mito-Y^. As mitochondrial DNA (mtDNA) copy number analysis did not indicate differences in mitochondrial quantity (Fig. 2a), we postulated that mitochondrial function may distinguish these cells. Therefore, we next analysed mitochondrial morphology and the structure of cristae using transmission electron microscopy (TEM) of ISCs. In line with the mtDNA analysis, the total mitochondrial area per cell was not different (Extended Data Fig. 3a) but the mitochondria in ISC^mito-O^ were more fragmented (Extended Data Fig. 3b–d). Moreover, ISC^mito-O^ had slightly lower cristae density, suggesting less oxidative phosphorylation (Fig. 2b). As the limited number of ISC^mito-O^ did not allow for reliable respirometry analysis (data not shown), and fluorescence probes for mitochondrial membrane potential and superoxide detection did not indicate differences in respiratory processes (Extended Data Fig. 3e,f), we analysed the metabolome of ISCs sorted according to their mitochondrial age composition (Extended Data Fig. 5g). Among the eight significantly differing metabolites, we found nicotinamide adenine dinucleotide (NAD)^+^, a metabolite with multiple roles in stem cell biology^23^, to be higher in ISC^mito-O^ (Extended Data Fig. 3g,h). However, as the NAD^+^ precursor nicotinamide riboside (NR) did not affect the regenerative capacity of organoids or PC emergence (Extended Data Fig. 3i,j), we did not pursue NAD^+^-related mechanisms further.

We next focused on the tricarboxylic acid (TCA) metabolite α-ketoglutarate (aKG), which curiously was higher in ISC^mito-O^ (Fig. 2c) despite mitochondrial morphology indicating lower oxidative phosphorylation^24^ (Fig. 2b and Extended Data Fig. 3b–d). Together with a modest reduction in succinate (Suc) (Fig. 2c), these data suggested that ISC^mito-O^ may uncouple TCA reactions from respiration. Therefore, we conducted metabolic tracing with ^13^C5-glutamine, which can be converted into aKG and enter the mitochondrial TCA cycle^25^ (Extended Data Fig. 5k). Metabolite tracing did not reveal notable differences in most TCA cycle metabolites, except for aKG, suggesting active maintenance of a higher aKG pool in ISC^mito-O^ (Extended Data Fig. 3l). Isotope-based metabolic labelling also enables tracing intermediates through multiple rounds of the TCA cycle, providing a proxy measurement for the TCA cycle rate (Extended Data Fig. 3m). We found that the rate of TCA cycling was faster in ISC^mito-O^ as they accumulated aKG species, which result from multiple TCA cycles more rapidly than ISC^mito-Y^ (Fig. 2d). Taken together, our findings on the density of cristae and mitochondrial morphology, and the faster TCA cycle turnover (without noticeable changes in mitochondrial reactive oxygen species formation or membrane potential), suggest that mitochondrial function in ISC^mito-O^ is partially uncoupled from mitochondrial respiration and may instead favour the generation of intermediates for anabolic activity and for guiding other cellular programmes^26^.

**Extended Data Fig. 4:**
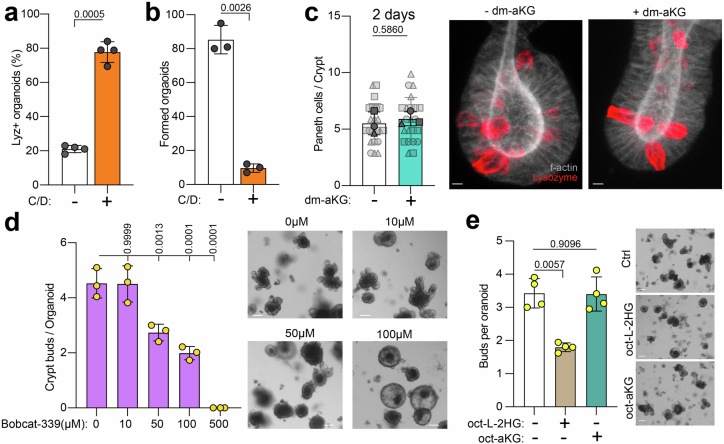
Notch and Tet inhibition. **a**, Paneth cells emergence after 48 h of Chir99021 and DAPT (C/D) supplemented niche-independent regenerative growth of ISCs. Two-tailed Student’s paired *t*-test (*n* = 4). Data mean ± SD. **b**, Niche-independent regenerative growth at day 6 of ISCs supplemented with Chir99021 and DAPT (C/D) for the first two days. Two-tailed Student’s paired *t*-test (*n* = 3). Data mean ± SD. **c**, Pulse (two days) treatment of organoids with dm-aKG does not increase Paneth cell number. Quantification and representative images at six days of culture (red lysozyme, white phalloidin). Scale bar, 5 μm. Two-tailed Student’s paired *t*-test (*n* = 3). Comparing organoids from three mice (indicated with symbols). Data mean±S.D. individual organoids shown in opaque. **d**, Left, Higher-dose Tet inhibitor treatment decreases organoid growth. Isolated crypts treated for 6 days with Bobcat-339. One-way ANOVA with two-tailed Dunnett’s post-hoc test *(n* = 3) Data mean ± SD. Right, representative images of day six organoids. Scale bars 50 µm. **e**, Regenerative organoid growth (crypt bud formation) is reduced by Octyl-L-2HG but not Octyl-aKG supplementation for 4 days. Two-tailed Student’s paired *t*-test (*n* = 4). Scale bars 50 µm. Source data

aKG is a TCA cycle metabolite with diverse functions in cellular metabolism and epigenetics^27^. Strikingly, cell-permeable dimethyl aKG (dm-aKG) increased niche-independent organoid formation by ISC^mito-Y^ to a level comparable with ISC^mito-O^ (Fig. 2e); the increase in organoid formation coincided with increased ability to recreate the PC niche (Fig. 2f). Interestingly, while dm-aKG also further increased PC emergence in ISC^mito-O^, it did not lead to increased organoid formation (Fig. 2e,f). This may represent an excessive investment into the PC lineage at the cost of self-renewal, which is similar to γ-secretase-inhibitor-induced excessive PC differentiation (Extended Data Fig. 4a,b). Finally, prolonged but not transient dm-aKG supplementation increased the number of PCs in mature organoids (Fig. 2g and Extended Data Fig. 4c), indicating that aKG promotes fate determination towards PCs. Taken together, these data indicated that the high aKG generated by old mitochondria promoted niche independence via PC renewal.

aKG is an epigenetic cofactor that affects differentiation of pluripotent stem cells^28^ and drives the expression of differentiation-associated genes in intestinal organoids with oncogenic mutations^29^. aKG levels regulate histone and DNA demethylation through aKG-dependent dioxygenase enzymes, Jumonji C (JmjC)-domain-containing lysine demethylases and ten-eleven translocation (TET) enzymes, respectively^27^. In the intestine, stem cells and progenitors show similar levels of histone marks and comparable chromatin states throughout their differentiation trajectory^30^. In contrast, DNA methylation (5-methylcytosine (5mC)), and especially TET-mediated 5-hydroxymethylcytosine (5hmC), exhibit dynamic alterations between stem cells and differentiated progeny^31^. Therefore, we explored whether the high aKG of ISCs^mito-O^ acts via TET-mediated 5-hydroxymethylation of DNA.

ISCs^mito-O^ had consistently higher 5hmC nuclear levels compared to ISCs^mito-Y^ from the same animal (Fig. 2h). Therefore, we next inhibited TET function with the cytosine-based small molecular inhibitor Bobcat339 (ref. ^32^). As loss of *Tet1* is detrimental to ISC function^33^, we determined a dose of Bobcat339 that had no effect on homeostatic organoid growth (Extended Data Fig. 4d) and assessed its impact on PC emergence. A low dose of Bobcat339 nullified the dm-aKG-induced PC emergence, while Bobcat339 alone showed no effect (Fig. 2i). Moreover, TET inhibition attenuated the PC emergence of ISCs^mito-O^ while having no effect on ISCs^mito-Y^ (Fig. 2j). Taken together, these data showed that increased aKG in ISCs^mito-O^ directed ISC fate towards generation of new PCs via TET activity.

**Extended Data Fig. 5:**
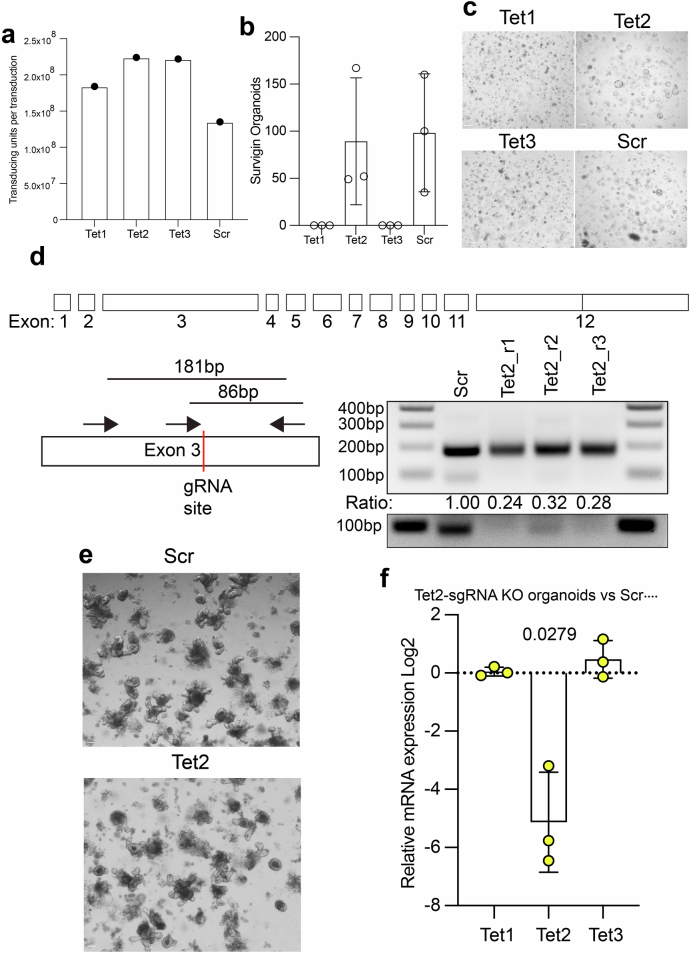
Lentiviral mediated generation of CrisprCas9 single guide knockout of Tets. **a**, Lentiviral transducing units used for the transduction with each guide based on p24 ELISA. **b**, Only Tet2 and Scramble guides generated surviving organoids two days post transduction, (before selection was started) Data mean ± SD, n = 3 independent transductions on independent mice. **c**, Representative brightfield images of organoids two days post transduction (before selection was started), scale bar 20 µm. **d**, Top, Schematic of Tet2 gene exons. Bottom, Strategy for PCR based validation of genetic targeting and editing. Two primers flanking the PAM site generating a 181 bp product, and one primer where 3’-end overlaps with the cut-site. Representative PCR results showing a reduction in gRNA-site primer 86 bp product. Repeated independently three times, once for each biological replicate used to create the sg-Tet2KO organoid lines. **e**, Representative brightfield images of Scr and Tet2 KO organoids grown and passaged for three weeks post selection, scale bar 50 µm. Repeated independently three times **f**, qPCR analysis of Tet1,2,3 in sg-Tet2KO organoids vs Scramble. Log_2_ normalized to mean of n = 3 Scramble. Data mean ± SD, n = 3 independent transductions of 3 independent mice two-tailed student’s unpaired *t*-test. Source data

TET activity is boosted by aKG and inhibited by other metabolites like Suc and 2-hydroxyglutarate (2HG)^34,35^. As 2HG was slightly decreased in ISCs^mito-O^ (Fig. 2k and Extended Data Fig. 5g), we treated organoids with cell-permeable octyl-l-2HG or octyl-aKG (octyl-based cell-permeable aKG as control) for 5 days. Octyl-l-2HG decreased the formation of new crypt buds while octyl-aKG did not (Extended Data Fig. 4e). Moreover, opposite to the effect of aKG, octyl-l-2HG reduced PC emergence (Fig. 2l), demonstrating that the balance of these two TET-influencing factors controls PC differentiation.

Mammals have three TET enzymes with partly overlapping targets and expression patterns. To elucidate which TETs confer the effect of aKG on PC emergence, we targeted *Tet1*, *Tet2* and *Tet3* with lentiviral CRISPR KO in organoids. We were unable to generate *Tet1* KO organoids (Extended Data Fig. 5a–c), which is in line with its critical function in the intestine^33^. More surprisingly, *Tet3* KO organoids were also not viable (Extended Data Fig. 7a–c), potentially reflecting the role of *Tet3* in the stress response^36^, although *Tet3* deletion only had a modest impact in vivo^37^. *Tet2* KO organoids were viable and had normal morphology (Extended Data Fig 5d–f); importantly, *Tet2* KO ISCs (Fig. 2m) demonstrated a lower ability to generate PCs (Fig. 2m). Moreover, the ability of aKG to substantially increase PC emergence was lost in *Tet2* KO cells (Fig. 2m). Taken together, these data showed that the levels of *Tet*-activating aKG and *Tet*-inhibiting 2HG, act as a metabolic lever guiding intestinal cell fate at least partially via *Tet2*.

**Fig. 3:**
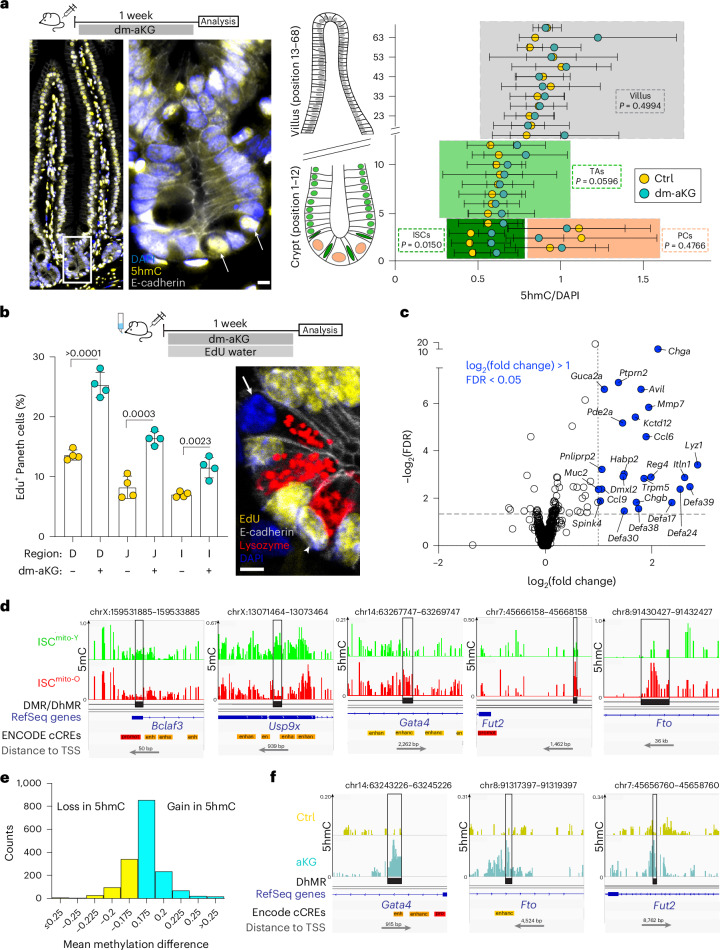
aKG-dependent methylome regulates PC renewal in vivo*.* **a**, Impact of in vivo dm-aKG on 5hmC in intestinal cells along the crypt–villus axis. Representative image of a control mouse indicating high 5hmC in PCs (arrow). DAPI, blue; 5hmC, yellow; E-cadherin, grey. dm-aKG treatment increased nuclear 5hmC significantly in ISCs. A two-tailed, unpaired Student’s *t*-test (*n* = 4) was used. Data are shown as the mean ± s.d. **b**, Impact of in vivo dm-aKG on PC renewal. Cells that renewed within a week are EdU^+^ (arrowhead); pre-existing cells older than a week are EdU^−^ (arrow). dm-aKG promoted PC renewal in all sections of the small intestine (D, duodenum; J, jejunum; I, ileum). A two-tailed, unpaired Student’s *t*-test (*n* = 4) was used. Data are shown as the mean ± s.d. Representative image of control crypt (blue, DAPI; red, lysozyme; yellow, EdU; white, E-cadherin). **c**, Volcano plot of transcriptomic changes in ISCs after 5 days of dm-aKG treatment (the blue data points are significant; false discovery rate (FDR) < 0.05, log_2_(fold change) > 1) **d**, Five representative hits of oxidative whole-genome bisulfite sequencing (WGBS-seq) differentially methylated or hydroxymethylated regions associated with PC or ISC function between ISCs^mito-O^ (red track) and ISCs^mito-Y^ (green track). **e**, dm-aKG induced more gains than losses of DhMRs in ISCs, as seen using E5hmC-seq. The bar graph shows the DhMR count for the indicated interval of hydroxymethylation difference. **f**, dm-aKG induced increased hydroxymethylation of WGBS-seq PC-associated and ISC-associated hits (*Fto*, *Gata4* and *Fut2*), as seen with the E5hmC-seq control (yellow track) and dm-aKG-treated ISCs (teal track). TSS, transcription start site. Scale bar, 5 µm (**a**,**b**). Source data

**Extended Data Fig. 6:**
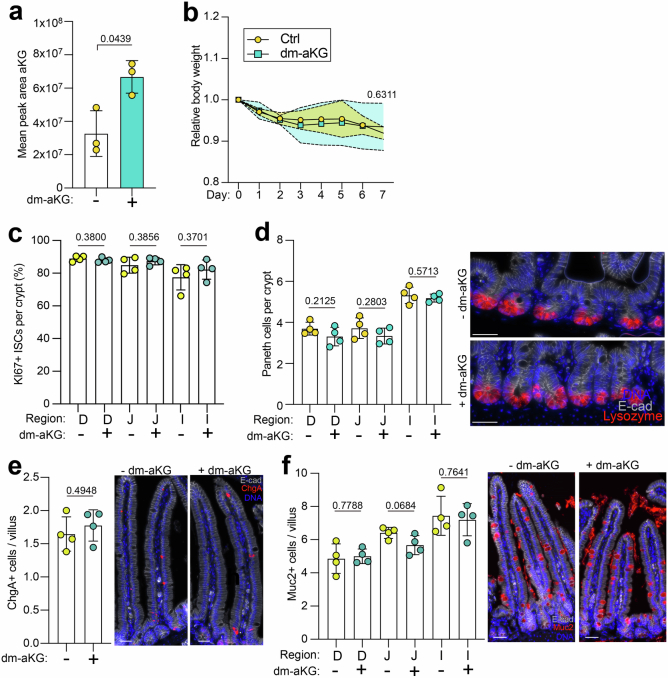
Characterisation of dm-aKG effects *in vivo.* **a**, dm-aKG treatment for one week increases total aKG (LC–MS mean peak area) in the intestine (*n* = 3) two-tailed student’s paired *t*-test. Data mean ± SD. **b**, Relative weight change of mice during dm-aKG treatment for one week. Daily data points represent mean and coloured area between dashed lines show the 50% interquartile range). Two-tailed Student’s unpaired *t*-test at 7 days *(n* = 4). **c**, Proliferative cells (ki67+) per crypt is unchanged by dm-aKG. two-tailed Student’s unpaired *t*-test (*n* = 4). Data mean ± SD. **d**, Left, Paneth cell number is unchanged across the intestine after dm-aKG treatment for one week (D=duodenum, J=jejunum, I=Ileum). Two-tailed Student’s unpaired *t*-test (*n* = 4). Data mean ± SD. Right, representative images of ileal crypts (blue=DAPI, red=lysozyme, white=E-cadherin) Scale bar 50 µm. **e**, Left, Enteroendocrine cell number is unchanged after dm-aKG treatment for one week. Two-tailed Student’s unpaired *t*-test (*n* = 4). Data mean ± SD. Right, representative images of jejunal villi (blue=DAPI, red=Chromogranin A, white=E-cadherin) Scale bar 50 µm. **f**, Left, Goblet cell number is unchanged across the intestine after dm-aKG treatment for one week (D=duodenum, J=jejunum, I=Ileum). Two-tailed Student’s unpaired *t*-test (*n* = 4). Data mean ± SD. Right, representative images of jejunal villi (blue=DAPI, red=Mucin 2, white=E-cadherin) Scale bar 50 µm. Source data

We next addressed the impact of aKG in vivo. Treatment of mice with dm-aKG administered intraperitoneally for 7 days increased aKG levels in the intestinal epithelium (Extended Data Fig. 6a) without general adverse effects (Extended Data Fig. 6b). To assess possible effects on methylation, we analysed 5hmC levels in tissue sections. Control mice showed an interesting pattern of nuclear 5hmC, where 5hmC gradually increased along the crypt–villus axis, with the exception of PCs displaying high 5hmC in the crypt (Fig. 3a). Importantly, dm-aKG treatment substantially increased 5hmC in ISCs but had no effects in other cell types (Fig. 3a). To probe the possible effects of dm-aKG induced 5hmC on ISC differentiation in vivo, we assayed the number of ISCs and PCs per crypt and the number of enteroendocrine and goblet cells per villus but noted no change after the 7-day treatment (Extended Data Fig. 6c–f). As the stem cell:PC ratio is tightly regulated^6^, and PCs have a particularly slow turnover rate^38^, we also analysed the renewal rate of PCs in vivo by supplementing drinking water with EdU over the course of the dm-aKG treatment. Strikingly, dm-aKG treatment increased the number of newly made PCs, thus promoting renewal of the epithelial ISC niche (Fig. 3b).

**Extended Data Fig. 7:**
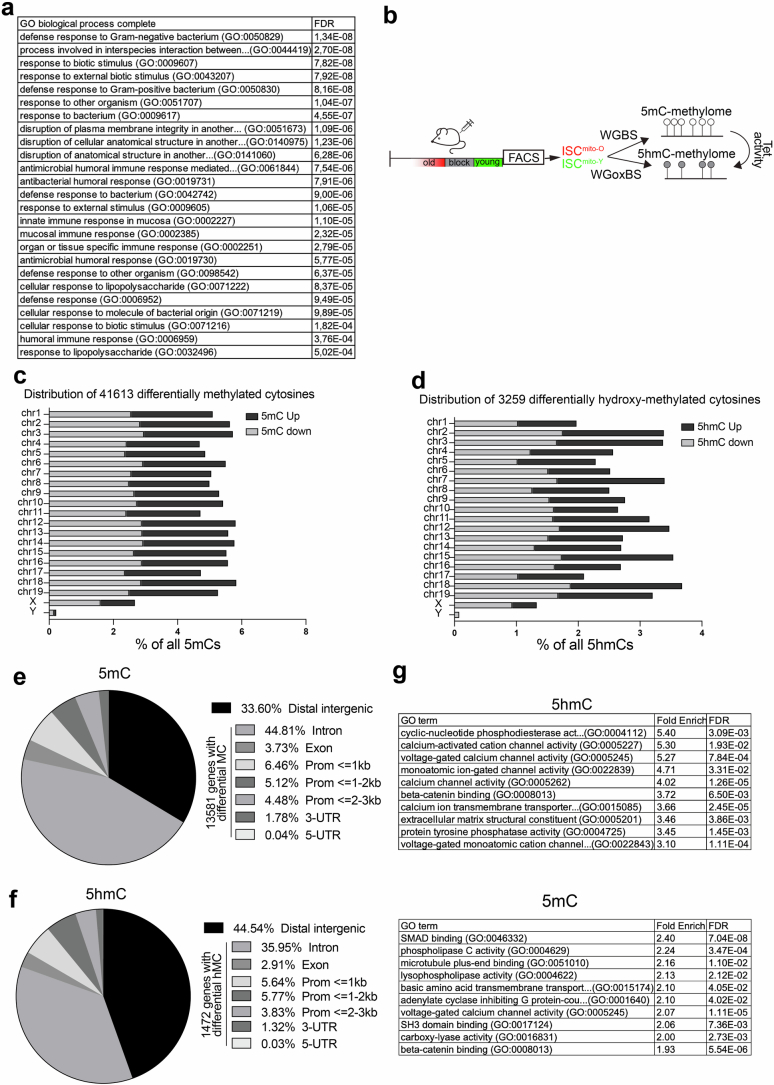
Mitochondrial age induced transcriptome and methylome analysis. **a**, Unbiased GO enrichment analysis of gene expression changes induced by dm-aKG treatment in ISCs. **b**, Schematic of WGoxBS-Seq experimental flow form Snap labelled cells. **c**, Genome wide distribution of differentially methylated cytosines between ISCs^mito-O^ and ISCs^mito-Y^. **d**, Genome wide distribution of differentially hydroxymethylated cytosines between ISCs^mito-O^ and ISCs^mito-Y^. **e**, Distribution of differentially methylated cytosines between ISCs^mito-O^ and ISCs^mito-Y^ across genomic elements. **f**, Distribution of differentially hydroxymethylated cytosines between ISCs^mito-O^ and ISCs^mito-Y^ across genomic elements. **g**, Unbiased GO enrichment analysis of differentially methylated (left) and hydroxymethylated (right) cytosines between ISCs^mito-O^ and ISCs^mito-Y^ showing top ten most enriched. Source data

**Extended Data Fig. 8:**
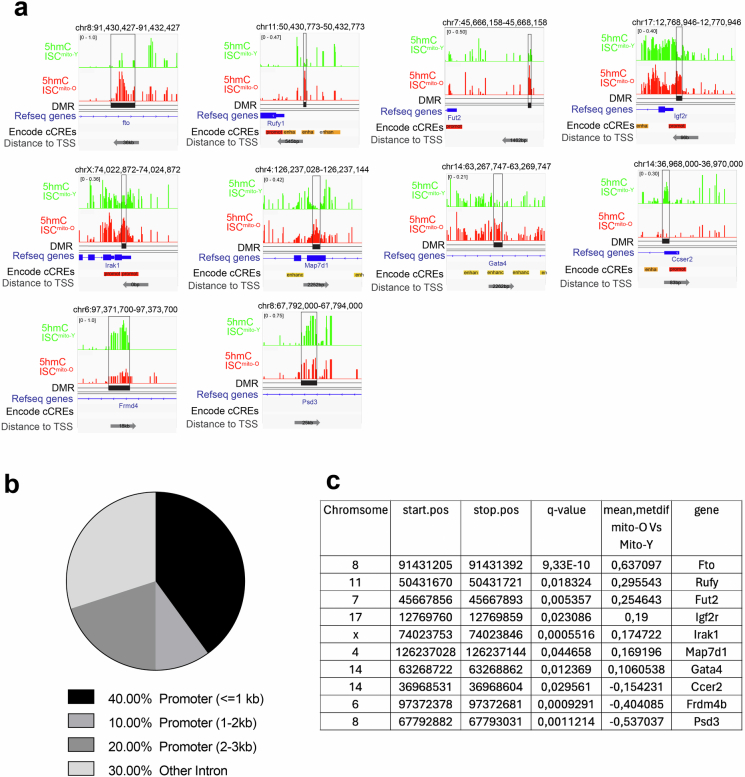
DhMR analysis of WGoxBS-Seq from ISCs^mito-O^ and ISCs^mito-Y^. **a**, 10 significant DhMRs between ISCs^mito-O^ (Red-track) and ISCs^mito-Y^ (Green-track). Annotated to the genome and Encode Ccre regulatory elements. **b**, The genomic element distribution of DhMRs between ISCs^mito-O^ and ISCs^mito-Y^. **c**, A table of significant DhMRs. Source data

**Extended Data Fig. 9:**
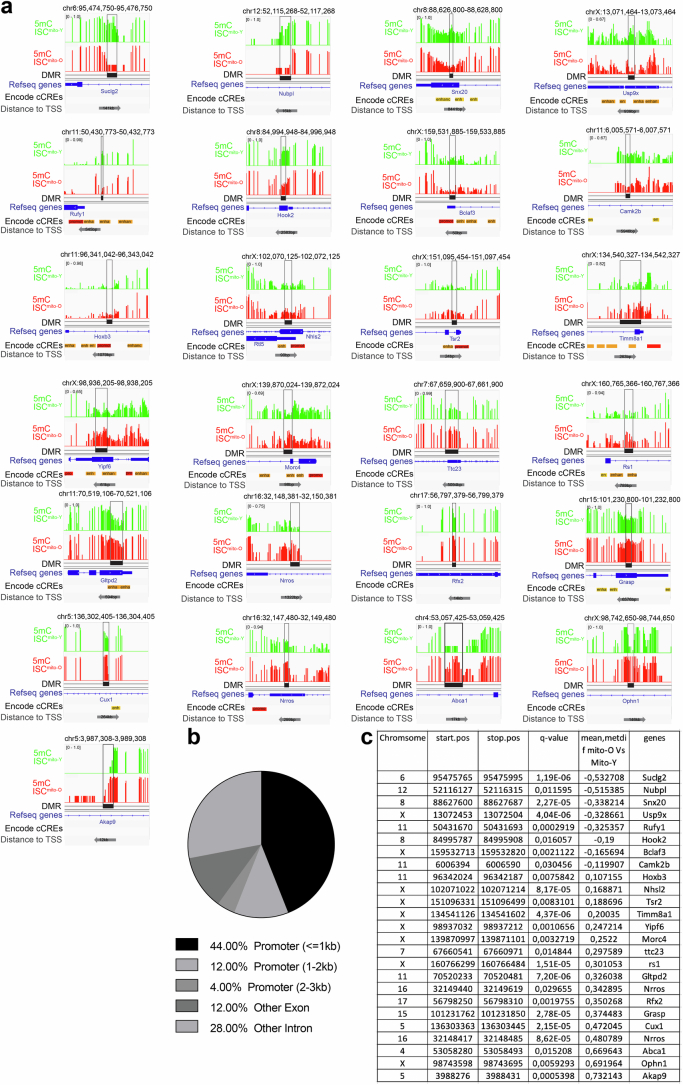
DMR analysis of WGoxBS-Seq from ISCs^mito-O^ and ISCs^mito-Y^. **a**, 25 significant DMRs between ISCs^mito-O^ (Red-track) and ISCs^mito-Y^ (Green-track). Annotated to the genome and Encode Ccre regulatory elements. **b**, The genomic element distribution of DMRs between ISCs^mito-O^ and ISCs^mito-Y^. **c**, A table of significant DMRs. Source data

As dm-aKG drove stem cells towards PC generation, we conducted mRNA sequencing of ISCs after a 7-day dm-aKG treatment. We found a set of 25 differentially expressed genes (Fig. 3c) with a striking upregulation of genes associated with innate immunity and secretory cell function (Extended Data Fig. 7a), which is consistent with a dm-aKG-driving PC fate. As the week-long dm-aKG treatment was expected to also induce secondary alterations, we next aimed to elucidate the early *Tet*-mediated epigenetic changes that are induced by the asymmetric apportioning of mitochondria and analysed the genome-wide DNA methylome (5mC) and hydroxymethylome (5hmC) in ISC^mito-O^ and ISC^mito-Y^ (Extended Data Fig. 7b). To identify potentially gene-regulating methylation events from genome-wide individual cytosine changes (Extended Data Fig. 7c–g), we focused on regions that contained at least five differentially methylated cytosines within 300 bp (5mC) or 500 bp (5hmC) located within genes and promoters (±3 kb). This revealed ten differentially hydroxymethylated regions (DhMRs) and 25 differentially methylated regions (DMRs) (Extended Data Figs. 8a and 9a). Importantly, a striking five of the 14 genes that had changes consistent with *Tet* activity (seven increased in 5hmC and eight decreased in 5mC) had links to intestinal stemness or secretory/PC differentiation, including *Gata4* (ref. ^39^), *Bclaf3* (ref. ^40^), *Fto*^41^, *Fut2* (ref. ^42^) and *Usp9x*^43^ (Fig. 3d).

**Extended Data Fig. 10:**
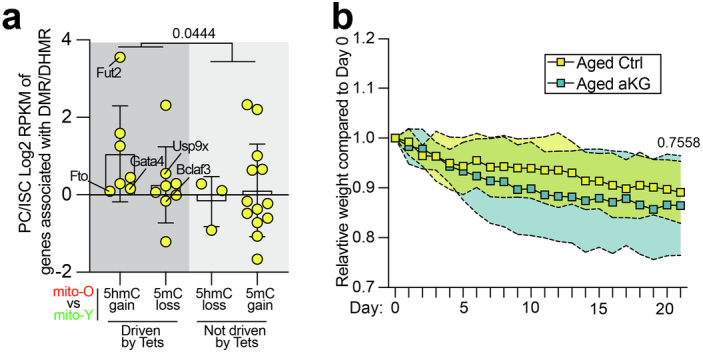
Dm-aKG induced niche renewal in aged mice. **a**, Paneth cell/ISCs gene expression (mean RPM) difference (Log2Fold) (Y-axis)) of genes that gained or lost 5mC and 5hmC between ISCs^mito-O^ and ISCs^mito-Y^ (X-axis). Data mean ± SD Kolmogorov-Smirnov Test (KS Test) comparing the distribution of DMRs driven by Tets (5hmC^gain^;5mC^loss^) and DMRs not driven by Tets (5hmC^loss^;5mC^gain^). **b**, Relative weight changes of aged (≥18 months) mice during once-daily dm-aKG treatment for three weeks. Daily data points represent mean and coloured area between dashed lines show the 50% interquartile range). Two-tailed Student’s unpaired *t*-test (*n* = 3) at 21 days. Source data

To understand which of the changes in 5hmC are attributable to aKG, we analysed the hydroxymethylome of ISCs upon aKG treatment. To overcome the technical limitations of bisulfite sequencing, where 90% or more of the DNA is destroyed, we instead used the recently developed enzymatic 5hmC sequencing (E5hmC-seq)^44^. Seventy percent of DhMRs (1,520 genes) we detected with enzymatic 5hmC-seq were consistent with aKG-driven *Tet* activity (Fig. 3e), including three of five genes we had identified from the ISC^mito-O^ methylome (Fig. 3f, compared to 3d). Finally, to gain insights as to whether the identified candidates represent possible factors initiating PC differentiation, or are markers of mature PCs, we compared the mRNA expression of the identified DMR-associated and DhMR-associated genes in ISCs and PCs. While genes associated with *Tet* activity had in general higher mRNA expression in PCs (Extended Data Fig. 10a), of the five candidates with reported links to the intestine, only *Fut2* with a microbiome-modulating function^45,46^ was substantially higher in PCs. Taken together, these data suggest that aKG from old mitochondria induces *Tet*-mediated epigenetic changes that potentially have a role in the early steps of fate determination.

**Fig. 4:**
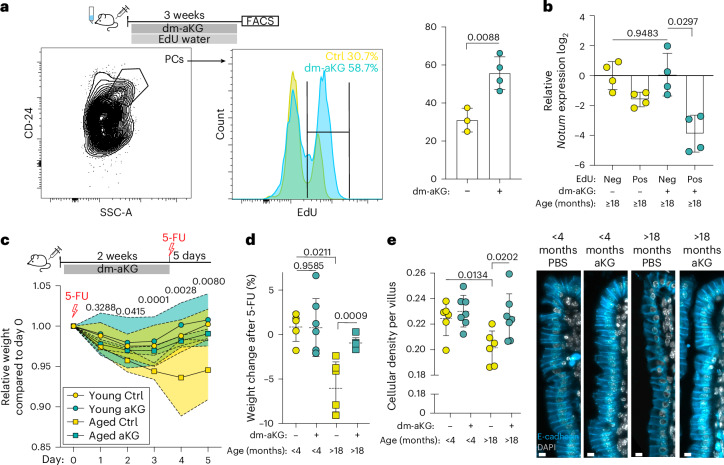
In vivo dm-aKG promotes regeneration of the aged intestine by inducing PC renewal. **a**, Once daily dm-aKG treatment for 3 weeks increased the percentage of renewed PCs in aged (≥18-month-old) mice. Representative FACS plot for sorting PCs (left, 2% contour plot) and EdU^+^ analysis of sorted PCs (middle, histogram). Plot of the flow cytometry analysis of EdU^+^ PCs. A two-tailed, unpaired Student’s *t*-test (PBS, *n* = 3; aKG, *n* = 4) was used. Data are shown as the mean ± s.d. **b**, *Notum* expression in renewed (EdU^+^) and pre-existing (EdU^−^) PCs of aged (≥18-month-old) mice after 3 weeks of dm-aKG. A two-tailed, paired Student’s *t*-test (*n* = 4) was used. Data are shown as the mean ± s.d. Data were normalized to the dm-aKG^−^ EdU^−^ population. **c**, Relative body weight of aged (≥18-month-old) and young (3–4-month-old) mice treated once daily with dm-aKG or vehicle for 2 weeks, followed by a single 5-FU (100 mg kg^−^^1^) injection. The daily data points represent the mean. Young (3–4-month-old, circles) and old (≥18-month-old, squares) mice are shown; the coloured area between the dashed lines represents the 50% interquartile range. The daily weight of aged dm-aKG-treated mice was compared to aged controls with a two-tailed, unpaired Student’s *t*-test (*n* = 7). **d**, Prophylactic dm-aKG treatment reduced 5-FU-induced weight loss at day 5 after injection in aged (≥18-month-old) mice without adverse effects on young (4-month-old) mice. A two-tailed, unpaired Student’s *t*-test (*n* = 6) was used. Data are shown as the mean ± s.d. **e**, Left: quantification of cellular density in ileal villi (cells per µm). Data are shown as the mean ± s.d.; each data point shown represents a mouse. A two-tailed, unpaired Student’s *t*-test (*n* = 6) was used. Right: representative immunofluorescence staining of villi after 5-FU treatment for 5 days (grey, DAPI; blue, E-cadherin). Scale bar, 10 μm (**e**). Source data

Renewal of the intestinal epithelium slows down with age^47^, and the coinciding increase in PC numbers^47,48^ suggests defects in normal niche renewal. We recently found that the regenerative capacity of the intestine declines during ageing as PCs begin to produce the secretory Wnt inhibitor *Notum*^48^. Therefore, increasing PC turnover with aKG may provide a strategy for replacing *Notum*-producing PCs with new cells that may express less *Notum*. Accordingly, dm-aKG treatment increased the PC renewal rate also in aged animals without adverse effects (Fig. 4a and Extended Data Fig. 10b). Furthermore, *Notum* expression was notably downregulated specifically in the newly made EdU^+^ PCs but not in the non-renewed EdU^−^ cells (Fig. 4b).

Finally, to test whether the niche renewal induced by dm-aKG also promoted regeneration of aged intestine, we analysed the ability of aged animals to recover from 5-fluorouracil (5-FU) chemotherapy-induced mucositis that results in loss of body weight^49^. We treated mice with 100 mg kg^−1^ (body weight) of 5-FU, resulting in a loss of body weight that young mice fully recover from within 5 days, but aged animals do not^48^. Strikingly, when niche renewal was induced with dm-aKG for 2 weeks before 5-FU treatment, weight loss in aged animals was considerably reduced (Fig. 4c,d). Consistent with improved regeneration and in line with earlier work^50^, epithelial cell density recovered in dm-aKG treated aged mice at a rate comparable to young animals (Fig. 4e). These results indicate that regeneration of the aged intestine can be metabolically promoted by transiently inducing the replacement of the aged niche.

Cellular metabolism has a pivotal role during tissue regeneration and ageing^51^. However, the mechanisms of metabolic cell fate regulation and the origin of distinct metabolic cell identities have remained less clear. In this study, we show that the age composition of organelles forms a facet of cell fate regulation in tissues, and that ISCs contain previously unrecognized metabolic and functional heterogeneity resulting from their mitochondrial age composition.

While the metabolic subdivision we illuminated is generated by asymmetric cell divisions, our data also predict that such divisions may not be functionally deterministic. ISC^mito-O^ and asymmetric divisions represent more than 10% of events among all ISCs; thus, deterministic PC differentiation would result in PC turnover rates greatly exceeding the rate observed by us and others^38^. Our data rather suggests that asymmetric divisions create cells biased for PC differentiation, providing a starting point for other mechanisms^52,53^. Together with our data on long-term organoid cultures (Extended Data Figs. 3a and 4c), this suggests that the clonal dynamics of ISC^mito-O^ and ISC^mito-Y^ can be relatively similar during homeostasis, and thereby fit the models predicting that fate determination in the intestine results from secondary events downstream of exclusively symmetric divisions and neutral clonal competition^19,20^. Interestingly, a recent model allowing both division modes also predicted that asymmetric divisions contribute to intestinal homeostasis^54^.

Although the outcome of asymmetric divisions is non-deterministic, the potency of aKG to promote PC renewal in vivo makes it plausible that ISC^mito-O^ are the source of most new PCs and have an important role after catastrophic loss of niche homeostasis. Indeed, aKG treatment induced the expression of genes such as defensins and lysozyme that are functionally important for mature PCs, but such changes were not yet seen in ISCs^mito-O^ emerging from asymmetric divisions. Instead, we found the metabolism of ISC^mito-O^ to epigenetically change a surprisingly limited set of loci associated with genes with the potential to govern PC differentiation. In this regard, it is interesting how symmetry breaking by the emergence of the first PC is the defining step changing spheroidal growth of non-differentiating stem cells into organoid growth with normal differentiation^52^. Yet, we found that the first ISC^mito-O^ division in vitro segregated old mitochondria symmetrically (Fig. 1g). Interestingly, one of the genes with epigenetic changes consistent with high aKG was *Fto*, an aKG-dependent mRNA demethylase that promotes the stability of transcripts of *Yap1* (ref. ^55^). Importantly, the aforementioned symmetry breaking is attributed to hitherto unexplained variability in *Yap1*, with transient increase in *Yap1*-licensing *DLL1*-*Notch* differences and resulting in PC emergence^52^. Taken together, we hypothesize that the age-selective segregation of mitochondria that occurs in the asymmetric subset of ISC divisions, generates a metabolic bias between the two daughter cells, which could epigenetically prepare the daughter cell receiving old mitochondria for symmetry breaking.

The mouse model developed in this study will facilitate future studies probing whether organellar age elicits functional heterogeneity missed by single-cell RNA sequencing (RNA-seq) studies also in other tissue stem cell systems. Indeed, aKG is reduced with ageing^56^ and mitochondrial defects are a hallmark of ageing^57^, raising the possibility that mitochondria-derived aKG levels limit methylation dynamics and stem cell function during ageing in multiple tissues. Our discovery of aKG as a way to promote renewal of a specific cell type highlights the active role cellular metabolism has in cellular fate determination and suggests that selective replacement of a defect-bearing cell type by metabolic stem cell guidance may provide applicative opportunities.

## Methods

### Mice

Animal studies were approved by the National Animal Ethics Committee of Finland and conducted with the support of the HiLIFE Laboratory Animal Centre Core Facility, University of Helsinki, under institutional guidelines. The licence numbers covering all related work are ESAVI-7011-2019 and ESAVI/18179/2020. Mice were maintained in C57BL/6J (Inotiv) background and housed in specific-pathogen-free conditions in individually ventilated cages, with Aspen bedding and nesting material. The light–dark cycle was 12 h; temperature was maintained at 21–22 °C and humidity at 40–60%. Mice had ad libitum access to Teklad Irradiated Global 16% Protein Rodent Diet food (Teklad, catalogue no. 2916) and water. Mice older than 18 months were considered old; mice between 3 and 6 months were considered young. Mice of both sexes were used in all experiments; sex was considered during the experimental design to ensure balanced representation in all groups. The SNAPtag-omp25 mouse (Mouse Genome Informatics: 6466976) was generated by introducing a CAG-*loxP*-3*polyA-*loxP*- SNAPtag-PTS1 cassette into the first intron of *ROSA26* in reverse direction using homologous recombination. Mice were crossed with Lgr5-EGFP-IRES-creERT2 mice^4^. For in vivo labelling of mitochondrial age classes, mice were administered one dose of 80 mg kg^−1^ TMX (catalogue no. T5648, Sigma-Aldrich) via intraperitoneal injection. TMX was dissolved in ethanol at 100 mg ml^−1^ and diluted to a working concentration of 10 mg ml^−1^ in corn oil (Sigma-Aldrich). Mice were then subsequently injected with 15–30 nmol of SNAP-Cell substrates for old (SNAP-Cell 647-SiR, catalogue no. S9102S) and young (SNAP-Cell TMR-Star S9105S SNAP-Cell 430, catalogue no. S9109S) mitochondria or SNAP-Cell Blocking (SNAP-Cell Block, catalogue no. S9106S). SNAP-Cell substrates and block were resuspended in 10 µl of dimethyl sulfoxide and diluted 1:100 in PBS for use. Unless otherwise stated in the figure legends, SNAP labelling was performed according to the procedure in Extended Data Fig. 1f. For in vivo proliferation, mice were injected intraperitoneally with 10 mg kg^−1^ EdU (Sigma-Aldrich) in PBS 2 h before euthanasia, or supplemented with 0.2 mg ml^−1^ EdU in their drinking water. Dm-aKG (catalogue no. 349631, Sigma-Aldrich) was diluted to 100 mg ml^−1^ from which mice were injected intraperitoneally with 600 mg kg^−1^ of dm-aKG twice daily unless otherwise stated in the figure legend. 5-FU (Sigma-Aldrich) was reconstituted in dimethyl sulfoxide at 100 mg ml^−1^ and a single intraperitoneal injection at a dose of 100 mg kg^−1^.

### Isolation of mouse small intestinal crypts

Mouse small intestinal crypts were isolated by flushing small intestines with ice-cold PBS and opening longitudinally, rubbing to remove mucus. The intestine was cut into small fragments and incubated with three changes of 10 mM EDTA in PBS on ice for 1 h and 45 min. The epithelium was then detached using vigorous shaking. To enrich crypts, tissue suspension was filtered through a 70-μm nylon mesh. Enriched crypts were washed once with cold PBS and kept on ice for follow-up procedures.

### Organoid culture

Crypts were resuspended in Advanced DMEM/F12 (Gibco), plated (200 crypts per 20 μl drop of Matrigel) and overlaid with 300 µl of ENR medium (Advanced DMEM/F12(Gibco), 1× GlutaMAX (Gibco), 100 U ml^−1^ penicillin and streptomycin, 10 mM HEPES, 1× B-27 (Gibco), 1× N-2 (Gibco), 50 ng ml^−1^ mouse EGF (R&D Systems), 100 ng ml^−1^ noggin (PeproTech) and 500 ng ml^−1^ R-spondin 1 (R&D Systems); Y-27632 (10 μM) was added for the first 2 days of culture. When indicated, ENR medium was supplemented with 2 mM dm-aKG unless otherwise stated in the figure legend, 10–100 µM Bobcat339 (catalogue no. HY-111558A, MedChemExpress), 1 mM NR (MedKoo Biosciences), 500 µM Octyl-l-2-α-hydroxyglutarate (catalogue no. 16367, Cayman Chemical), and 500 µM Octyl-α-ketoglutarate (catalogue no. 2205, Sigma-Aldrich), as stated in the figure legends. The medium was changed every 2 days. Equal amounts of vehicle were used in the controls.

### Single-cell sorting and analysis

Single cells were isolated by dissociating crypts in TrypLE Express (Gibco) with 1,000 U ml^−1^ of DNase I (Roche) at 32 °C for 2 min. Cells were washed and stained with the following antibodies: CD31 PerCP-Cy5.5 (clone MEC13.3, BD Biosciences); CD45 PerCP-Cy5.5 (clone 30-F11, BD Biosciences); Ter119 PerCP-Cy5.5 (clone TER-119, BD Biosciences); CD326 BV786 (clone G8.8, BD Biosciences); CD24 BV421 (clone M1/69, BD Biosciences), all at 1:500 dilution. Finally, cells were resuspended in HBSS containing 0.2% BSA supplemented with 2 μg ml^−1^ 7-AAD (Thermo Fisher Scientific) for live cell separation. Cells were sorted using a FACSAria Fusion flow cytometer (BD Biosciences). Sorting strategies were: ISC, Lgr5-EGFP^hi^CD326^+^CD24medCD31^−^Ter119^−^CD45^−^7-AAD^−^; ISC^mito-O^, sir-647^hi^Tmr-star^med-lo^Lgr5-EGFP^hi^CD326^+^CD24medCD31^−^Ter119^−^CD45^−^7-AAD^−^; ISC^mito-Y^, sir-647^med-lo^Tmr-star^hi^Lgr5-EGFP^hi^CD326^+^CD24medCD31^−^Ter119^−^CD45^−^7-AAD^−^; PC, CD24^hi^SideScatter^hi^Lgr5-EGFP^−^CD326^+^CD31^−^Ter119^−^CD45^−^7-AAD^−^; transient amplifying cell, Lgr5-EGFP^lo^CD326^+^CD24medCD31^−^Ter119^−^CD45^−^7-AAD^−^; SNAP^+^ cell, sir647^+^CD326^+^CD31^−^Ter119^−^CD45^−^7-AAD^−^; and CD24^med^SSC^med^ stem-cell-enriched population from organoids, SSC^med^CD24^med^CD326^+^7-AAD^−^. For the mitochondrial membrane potential and superoxide detection analysis, singles cells were incubated with 20 nM tetramethylrhodamine (Thermo Fisher Scientific) or 5 µM superoxide Detection Reagent Orange (Enzo Life Sciences), respectively for 30 min at 37 °C. Gating strategies were as described above, except for young mitochondria being labelled with SNAP-Cell 430. For EdU analysis using FACS, single-sorted cells (gating strategies as described above) were fixed with 4% paraformaldehyde (PFA) for 10 min, after which they were washed twice and stained with the Click-iT kit (C10418, Sigma-Aldrich) and reanalysed for Click-iT EdU PacificBlue. Flow cytometry data were collected with FACSdiva (v.8) and analysed with Flow-Jo (v.10.8)

### Single-cell culturing

For the single-cell regeneration assay, equal numbers of ISCs and PCs were cultured alone (3,000 ISCs) or cocultured (3,000 ISCs + 3000 PCs) in ENR medium supplemented with an additional 500 μg ml^−1^ R-spondin 1 (to yield a final concentration of 1 μg ml^−1^), 100 ng ml^−1^ Wnt3A (R&D Systems) and 10 μM Jagged-1 peptide (AnaSpec) for the first 4 days; Y-27632 (10 μM) was added to the medium for the first 2 days. The number of organoids per well was quantified at day 6; organoid budding was quantified at 10 days. The medium was changed every 2 days. For the PC emergence assay from single-sorted cells, the same medium composition as described above was used; organoids were fixed with 4% PFA. When indicated, ENR medium was supplemented with 2 mM dm-aKG, 10 µM Bobcat339, 1 mM NR, 500 µM Octyl-l-2-α-hydroxyglutarate) and 500 µM Octyl-α-ketoglutarate. In Extended Data Fig. 4a,b, C/D refers to 10 µM (CHIR99021) and 10 µM (DAPT).

### CRISPR–Cas9 gene editing of intestinal organoids

Guide RNAs for the target gene KO^58^ were designed with the CRISPR design tool (https://chopchop.cbu.uib.no). Guides were cloned into lentiCRISPR v2 vector (catalogue no. 52961, Addgene). Lentiviral vectors were produced in 293FT cells (catalogue no. R70007, Thermo Fisher Scientific) and concentrated with Lenti-X concentrator (Clontech). The 293FT cell line was not authenticated in the laboratory, but tested negative for *Mycoplasma*. Cultured intestinal organoids were treated with 6 µM CHIR99021 for 4 days to enrich for stem cells. Organoids were mechanically disrupted and dissociated to small fragments with TrypLE Express supplemented with 1,000 U ml^−1^ DNase I for 5 min at 32 °C. Fragments were resuspended in transduction medium (ENR medium supplemented with 8 μg ml^−1^ polybrene (Sigma-Aldrich), 1 mM of nicotinamide, 6 µM CHIR99021, 10 μM Y-27632) and mixed with concentrated virus at 1 × 10^8^–2.5 × 10^8^ transducing units per transduction; the p24 lentivirus capsid protein concentration was determined using enzyme-linked immunosorbent assay (Biomedicum Virus Core). Samples were spinoculated for 1 h at 600*g* and 32 °C followed by 6-h incubation at 37 °C, after which they were plated in 60% Matrigel overlaid with transduction medium without polybrene; 2–3 days after transduction, infected clones were selected by adding 2 μg ml^−1^ puromycin (Sigma-Aldrich) to the medium. Four days after selection, clones that survived were expanded in normal ENR medium and clonogenic growth was assessed. KO was confirmed using three-primer PCR around the guide RNA target site and quantitative PCR. LentiCRISPR v2 was a gift from F. Zhang^59^. Oligonucleotides are listed in Supplementary Table 9.

### Immunohistochemistry and immunofluorescence

Tissues were fixed in 4% PFA. Antigen retrieval was performed by boiling in citrate buffer at pH 6 (Sigma-Aldrich) for 20 min. This was followed by permeabilization with 0.5% Triton X-100 (Sigma-Aldrich) for 20 min at room temperature. For EdU staining, we performed Click-IT chemistry. For 5hmC staining, permeabilization was followed by denaturing of DNA with 4N HCl for 30 min at room temperature, followed by washing once with distilled water and neutralization with 100 mM Tris-HCl, pH 8.5, for 15 min at room temperature. The primary antibodies lysozyme (EC3.2.1.17, 1:500 dilution, Dako), E-cadherin (1:500 dilution, catalogue no. 610181, BD Biosciences), chromogranin A (1:500 dilution, ab15160, Abcam), Mucin2 (1:500 dilution, catalogue no. sc-15334, Santa-Cruz Biotechnology) and 5hmC (1:500 dilution, catalogue no. AB_10013602, Active Motif) were incubated over night at +4 °C and detected with Alexa Fluor 488/594/633/647-conjugated anti-rabbit, anti-rat or anti-mouse secondary antibodies (1:500 dilution, Thermo Fisher Scientific) using a 1-h incubation at room temperature. Nuclei were co-stained with DAPI (1 μg ml^−1^, Thermo Fisher Scientific) or Hoechst 33342 (1 μg ml^−1^, Thermo Fisher Scientific). For cellular frequency quantification in crypts from tissue sections, only crypts with an uninterrupted lumen from crypt bottom to crypt neck were used for quantification. For organoids, fixing was directly preceded by permeabilization and followed by blocking and primary antibody incubation. Organoid cell borders were visualized by Phalloidin-Atto 565 (1:500 dilution, catalogue no. 65906, Sigma-Aldrich). Images were acquired with a Leica TCS SP8 STED 3X CW 3D confocal microscope with HC PL apochromatic (APO) ×10/0.40 CS2 air (working distance (WD) = 2.56 mm) HC PL APO ×20/0.75 IMM CORR CS2 water (WD = 0.66 mm), HC PL APO ×63 water (numerical aperture (NA) = 1.20), motCORR CS2 objective and HC PL APO ×93/1.30 motCORR STED WHITE glycerol (WD = 0.3 mm) and LAS X (v.5), and anlaysed with Fiji (v.2). For Figs. 3a,b and 4e, and Extended Data Fig. 6d–f, slides were scanned with Pannoramic 250 FLASH II (3DHISTECH) using a ×40/0.95 air objective and analysed with Slideviewer (v.2).

### Live organoid imaging

Organoids were grown until day 6 on coverglass-bottomed Mattek dishes. SNAP-Cell substrate was added in ENR medium for 30 min followed by washing and incubation with fresh ENR medium for 2 h before imaging. Then, 100 nM MitoTracker green FM (Thermo Fisher Scientific) was added to the ENR medium for 30 min and washed once before imaging. Images were acquired using a Leica TCS SP8 STED 3X CW 3D confocal microscope with HC PL APO ×63 water (NA = 1.20) motCORR CS2 objective.

### Whole-mount staining

Small intestinal tissue was flushed with PBS and fixed for 4 h at room temperature with 4% PFA followed by washing and overnight clearing in 80% glycerol containing 2 µg ml^−1^ DAPI. Tissues were imaged using a Leica TCS SP8 STED 3X CW 3D confocal microscope with HC PL APO ×20/0.75 IMM CORR CS2 water (WD = 0.66 mm), HC PL APO ×63 water (NA = 1.20) motCORR CS2 objective and HC PL APO ×93/1.30 motCORR STED WHITE glycerol (WD = 0.3 mm).

### Immunocytochemistry

Sorted cells were centrifuged with a Shandon Cytospin 4 (Thermo Fisher Scientific) for 5 min at 600*g* followed by air-drying overnight or allowed to settle on 0.01% poly-l-lysine-coated (catalogue no. A-005-C, Merck Millipore) coverglass-bottomed Mattek dishes for 30 min at 37 °C followed by fixation with 4% PFA and immunostaining with 5hmC or Hoechst 33342.

### Asymmetric inheritance of mitochondria

Division pairs were quantified using ImageJ. All the stacks were Z-projected with sum intensity, Cell borders were identified using brightfield microscopy and the shape of mitochondrial networks. The total intensity of young or old mitochondria in two daughter cells was considered 100% and data were plotted as a proportion of the total 100% of mitochondria of that age.

### Metabolomics

For the isotopic tracing experiments, isolated single cells were incubated for 30 min with 2 mM l-glutamine-^13^C_5_ (catalogue no. 605166, Sigma-Aldrich) in EN medium. Cells were washed in PBS and metabolites were extracted in ice-cold 80% acetonitrile and vortexed for 5 s. Samples were centrifuged at 15,800*g* for 10 min at 4 °C and the supernatant was stored in liquid nitrogen. For tissue extraction, samples were snap-frozen in liquid nitrogen and ground in a precooled pestle and mortar with liquid nitrogen; powdered tissue was moved into an extraction tube and four volumes of −20 °C extraction buffer was added and incubated on ice for 30 min; this was followed by centrifugation at 15,800*g* for 10 min at 4 °C, and the supernatant was stored in liquid nitrogen. Samples were analysed on a Thermo Q Exactive Focus Quadrupole Orbitrap mass spectrometer coupled with a Thermo Dionex UltiMate 3000 HPLC system (Thermo Fisher Scientific). The HPLC was equipped with a hydrophilic ZIC-pHILIC column (150 × 2.1 mm, 5 μm) with a ZIC-pHILIC guard column (20 × 2.1 mm, 5 μm, Merck Millipore). A 5-μl sample was injected into the LC–MS instrument after quality control in randomized order, with every tenth sample as a blank. A linear solvent gradient was applied in decreasing organic solvent (80–35%; 16 min) at a 0.15 ml min^−1^ flow rate and 45 °C column oven temperature. Mobile phases were carried out in aqueous 200 mmol l ^−1^ ammonium bicarbonate solution (p9.3, adjusted with 25% ammonium hydroxide), 100% acetonitrile and 100% water. The ammonium bicarbonate solution was kept at 10% throughout the run. Metabolites were analysed using a mass spectrometer with a heated electrospray ionization source, using polarity switching and the following settings: resolution of 70,000 at *m/z* = 200; spray voltages of 3,400 V for positive and 3,000 V for negative modes; sheath gas of 28 a.u. and auxiliary gas of 8 a.u.; vaporizer temperature of 280 °C; and ion transfer tube temperature of 300 °C. The instrument was controlled using Xcalibur v.4.1.31.9 (Thermo Fisher Scientific). Metabolite peaks were confirmed using commercial standards (Sigma-Aldrich). Data quality was monitored throughout the run using an in-house quality control cell line. After final peak integration with the TraceFinder v.4.1 SP2 software (Thermo Fisher Scientific), peak area data were exported as Excel files. Metabolite peak areas were corrected for background by subtracting the peak areas observed in an extraction buffer sample; the corrected values were normalized to the cell number. All metabolite peak areas are listed in Supplementary Table 2.

### ISC^mito-O^ and ISC^mito-Y^ RNA-seq and data processing

Total RNA from sorted ISC^mito-O^ and ISC^mito-Y^ (*n* = 6) was isolated with TRIzol. RNA was treated with HL-dsDNase (catalogue no. 80200-050, ArticZymes) to remove residual DNA. The NuGEN Ovation SoLo RNA-Seq kit was used for Illumina library preparation (NuGEN Technologies). Purified total RNA (10 ng) was used and primers for ribosomal removal were designed and used as outlined in the kit manual. Libraries were purified with AMPure XP beads (Beckman Coulter), and quantified and run on a NextSeq 500 Mid Output 150-cycle kit at a concentration of 1.25 pM. The sequencing read quality was checked with FastQC (v.0.11.8)^60^. The sequencing reads were mapped to the GRCm38 primary assembly genome using STAR (v.2.7.3a)^61^ with the GENCODE vM24 primary assembly annotation. Post-mapping sample quality was checked with RSeQC (v.3.0.1)^62^. Reads were deduplicated with UMI-tools (v.1.0.1)^63^, and transcript expression was quantified with kallisto (v.0.46.1)^64^ using a GENCODE vM24 transcript sequence index. The kallisto transcript abundance estimates were summarized into gene expression counts with tximport (v.1.14.0)^65^ and used with DESeq2 (v.1.26.0) in the differential expression analysis^66^. A paired design was used in the differential expression analysis by including a covariate for mouse in the linear model. Differentially expressed genes are listed in Supplementary Table 1. Gene set enrichment analysis was performed with the camera function of the limma package (v.3.42.0)^67,68^. The gene sets for the intestinal epithelial cell type markers were obtained from Haber et al.^18^; the *Mex3a*^+^ cell markers, the label retaining the cell signature genes and the intestinal bipotent progenitor markers were obtained from Barriga et al.^9^.

### ISC^mito-O^ and ISC^mito-Y^ oxidative WGBS-seq and data processing

Sorted ISC^mito-O^ and ISC^mito-Y^ from four mice were pooled per replicate to a total of four replicates (*n* = 4). Library preparation for oxidative WGBS-seq, post-processing of the raw data, data alignment and methylation calls were generated at the Epigenomics Core, Weill Cornell Medicine as follows: 5mC and 5hmC modifications were determined using oxidative WGBS-seq with the NuGEN Ultralow Methyl-Seq with TrueMethyl oxBs (catalogue no. M01512, Tecan). Approximately 300 ng genomic DNA were sonicated using a Covaris S220 ultrasonicator to a mean size of 250–300 bp. For quantitative assessment of oxidation, DNA was spiked with 1% of control DNA duplexes, containing C, 5mC and 5hmC bases at known positions, obtained from Cambridge Epigenetix. The sonicated DNA was split into two aliquots of 150 ng; one aliquot was oxidized and bisulfite-converted, the other was mock-oxidized before bisulfite conversion. Oxidative bisulfite libraries required ten PCR amplification cycles; bisulfite libraries required eight cycles. Library quality was assessed on an Agilent TapeStation D5000 (Agilent Technologies). The resulting libraries were normalized to 10 nM, pooled, clustered on a paired-end read flow cell and sequenced for 150 cycles on an Illumina NovaSeq 6000 sequencing system, obtaining about 300 million clusters per library. Primary processing of sequencing images was done using the Illumina Real Time Analysis software. Raw data were quality-controlled and aligned to the mouse genome build GRCm38.p6 (mm10); methylation calls were generated using the Epigenomics Core in-house bisulfite sequencing analysis pipeline^69^. Methylation sites with a minimum read coverage of ten were selected for the following analysis. 5-Hydroxymethylation was obtained by subtracting the oxidative bisulfite from the bisulfite frequencies. Methylation data for CpGs was processed using the methylKit (v.1.33.1) in R. Differentially methylated cytosines (DMCs) had a minimum methylation difference of 25% and a *q* cut-off of 0.01. All significant DMCs and DhMCs are listed in Supplementary Tables 3 and 4. Unbiased Gene Ontology (GO) term enrichment analysis was performed based on statistically significant DMCs and DhMCs associating with genes using PANTHERDB GO molecular function complete^70^. DMRs were obtained using metilene (v.0.2–8) as with enzymatic 5hmC, but with no estimation of missing data and with –M300 and –M500 for 5mC and 5hmC, respectively. DMRs were annotated using ChIPseeker with gene models from TxDb.Mmusculus.UCSC.mm10.knownGene. All significant DMRs and DhMRs are listed in Supplementary Tables 5 and 6. The PC versus ISC mRNA fold change of the DMRs associating with genes in ISC^mito-O^ was obtained from the RNA-seq data^48^ (ArrayExpress E-MTAB-7916).

### Dm-aKG-treated ISC RNA-seq and data processing

Total RNA from sorted ISCs from dm-aKG-treated or Ctrl mice (*n* = 6) were isolated with TRIzol. Total RNA was treated with DNase using the Heat&Run gDNA Removal kit (ArcticZymes) to remove residual DNA. From the resulting RNA, RNA-seq libraries were created using the Universal RNA-Seq with NuQuant kit (Tecan). Complementary DNA (cDNA) was generated using a mixture of random and poly(T) priming from 15–16 ng total RNA. Then, the cDNA was fragmented, followed by end-repair, adaptor ligation, strand selection and ribodepletion using AnyDeplete mouse probes (Tecan). The final library amplification step was performed with 18× cycles of PCR. Sample libraries were then pooled and converted to be compatible with the AVITI sequencer (Element Biosciences) using the Adept Rapid PCR-Plus protocol (Element Biosciences). The library was then sequenced with the AVITI Medium Output kit using 2× 75-bp reads. Sequences aligning to ribosomal RNA were removed using SortMeRNA (v.4.3.6)^71^. The remaining sequences were aligned to the GRCm39 mouse genome using STAR (v2.7.11a)^61^. Gene-level read counts were obtained using HTSeq (v.2.0.2), setting the minimum alignment quality to 20 (ref. ^72^). Differential expression analysis was performed with DESeq2 (v.1.40.2)^66^. Read counts were transformed using the variance stabilizing transformation in DESeq2; log fold values were shrunk with ashr (v.2.2-63)^73^. Differentially expressed genes are listed in Supplementary Table 7. Unbiased GO term enrichment analysis was performed based on statistically significant genes with a log_2_ fold change ≥1 (highlighted in Fig. 3c using PANTHERDB GO biological process complete^70^.

### Dm-aKG-treated ISC E5hmC-seq and data processing

ISCs from six PBS-treated and six dm-aKG-treated mice were sorted and total DNA was isolated using the Quick-DNA microprep kit (Zymo Research). DNA was fractionated using Bioruptor NGS (Diagenode) after adding control DNA from the E5hmC-seq kit (NEB-E3350S) to 250 ng of sample. The sonicated DNA libraries were constructed using NEBNext Primers for Epigenetics (Dual Index Primer Pairs Set 2B, catalogue no. E3392S). The sample libraries were pooled and converted to be compatible with the AVITI sequencer using the Adept Rapid PCR-Plus protocol. The library was then sequenced with the AVITI High Output kit using 2× 150-bp reads. Alignment of sequence data and the following methylation calls were performed with BiSulfite Bolt (v1.4.8)^74^ on the GRCm38.p6 (mm10) mouse genome. Methylation sites were called at a minimum read depth of three. To analyse DMRs, methylation sites were filtered to contain only those where at least five of six samples had non-missing values. Regions and their methylation levels were computed using metilene (v.0.2-8) with the following parameters: -d 0.15 -m 5 -X 5 -Y 5 -M 500 (ref. ^75^). DMRs are listed in Supplementary Table 8.

### qPCR with reverse transcription

RNA was isolated using TRIzol. For PFA-fixed samples, cells were pelleted and frozen in liquid nitrogen after which the RecoverAll Total Nucleic Acid Isolation Kit for FFPE (Thermo Fisher Scientific) was used, starting from protease digestion. Isolated RNA was transcribed with a cDNA synthesis kit using Oligo(dT) primers or random hexamers for the PFA-fixed samples (Molecular Probes). qPCR amplification was detected with the SYBRGreen (2× SYBRGreen mix, Applied Biosciences) method, using the CFX384 qPCR instrument (Bio-Rad Laboratories) and analysed with CFX Maestro (v.4.1) (Bio-Rad Laboratories). Samples were run as triplicates and genes of interest were normalized to *Actb*. For the pre-amplification of *Notum*, cDNA was pre-amplified for 14 cycles with the PerfeCTa PreAmp SuperMix (Quantabio) according to the manufacturer’s instructions. Nested primers were removed after pre-amplification with Thermolabile Exonuclease I (New England Biolabs) before performing qPCR with reverse transcription using the oligonucleotides listed in Supplementary Table 9.

### Mitochondrial DNA analysis

DNA was isolated from sorted cells using Quick-DNA microprep kit. The mitochondrial DNA in each sample was normalized to nuclear DNA using qPCR. Samples were run in triplicate. Oligonucleotides are listed in Supplementary Table 9.

### TEM

Sorted cells were attached to glass coverslips (thickness no. 1). Cells were fixed with 2% glutaraldehyde in 0.1 M sodium cacodylate buffer, pH 7.4, for 30 min at room temperature, post-fixed with 1% reduced OsO_4_ in the same buffer, for 1 h on ice, dehydrated through a series of ethanol and acetone, and embedded into Epon (TAAB 812 resin). Then, 60-nm-thin sections were cut using a Leica UCT6 microtome and post-stained with 0.5% uranyl acetate (SPI-Chem) and 3% lead citrate (Leica Microsystems). TEM imaging was done with a Jeol JEM-1400 transmission electron microscope (operated at 80kV, equipped with a Gatan Orius SC 1000B bottom-mounted charge coupled device camera. At least ten cells from four mice were analysed. The mitochondrial profile areas and density of cristae were analysed using Fiji (v.2).

### Statistical analysis

No statistical methods were used to predetermine sample sizes but our sample sizes are similar to those reported in previous publications^5,6,9^. Data collection and analysis were not performed blind to the conditions of the experiments. All images were analysed with Fiji (v.2). Microsoft Excel (v.16.68), Prism (v.9.4.1) and Integrative Genomics Viewer (v.2.6.12) were used for the statistical analysis and visualization of data. All data were analysed using two-tailed Student’s *t*-tests when comparing multiple groups, and a one-tailed ANOVA with Tukey’s post-hoc test or Dunnet’s post-hoc test, as shown in the figure legends. Exact *P* values are presented in the corresponding figures. A paired *t*-test was applied if the day of organoid growth quantification varied between pairs (samples processed the same day were paired) or if the phenotype after treatment was compared to the control from the same mouse (samples from the same mouse were paired). Whether a test was paired or unpaired is noted in the figure legends. *P* < 0.05 was considered significant. Data distribution was assumed to be normal but this was not formally tested. Mice were randomly assigned to experimental groups when applicable, but no additional randomization procedures were applied. No data points or animals were excluded from the analyses. Replicates in all experiments are biological replicates originating from separate mice. Data are shown as the mean ± s.d. with all individual data points showing.

### Sex

Both sexes were used throughout the study. Sex was considered during the experimental design to ensure balanced representation in all groups. Experimental replicates were always individual mice and cells were compared to cells originating from the same mouse in pair-wise fashion, intrinsically producing sex-matched comparisons.

### Reporting summary

Further information on research design is available in the Nature Portfolio Reporting Summary linked to this article.

## Supplementary information

Supplementary Information Supplementary Fig. 1.

Reporting Summary

Supplementary Tables 1–9 Table 1: RNA-seq of sorted ISCs enriched for young or old mitochondria. DESeq2 analysis of differentially expressed genes in ISCs (young mito versus old mito, that is, negative value higher in young mito, positive value higher in old mito) (*n* = 6 mice). Table 2: Mean peak areas corrected for the background of the identified metabolites in the metabolomics analysis of ISCs enriched for young or old mitochondria (*n* = 5 individual mice). Table 3: Oxidative WGBS. DhMCs between ISCs enriched for young or old mitochondria (young versus old mito) (that is, negative value higher methylation in young mito; positive value higher methylation in old mito). *n* = 4 biological replicates of pooled mice. Table 4: Oxidative WGBS. DMCs between ISCs enriched for young or old mitochondria (young versus old mito, that is, negative value higher methylation in young mito; positive value higher methylation in old mito). *n* = 4 biological replicates of pooled mice. Table 5: Oxidative WGBS. DhMRs between ISCs enriched for young or old mitochondria (young versus old mito, that is, negative value higher methylation in young mito; positive value higher methylation in old mito). *n* = 4 biological replicates of pooled mice. Table 6: Oxidative WGBS. DMRs between ISCs enriched for young or old mitochondria (young versus old mito, that is, negative value higher methylation in young mito; positive value higher methylation in old mito). *n* = 4 biological replicates of pooled mice. Table 7: RNA-seq of sorted ISCs from mice treated with dm-aKG or PBS. DESeq2 analysis of differentially expressed genes in ISCs (PBS versus dm-aKG, that is, negative value higher methylation in OBS; positive value higher methylation in dm-aKG-treated mice). *n* = 6 mice. Table 8: Oxidative WGBS. DhMRs between ISCs enriched for young or old mitochondria (young versus old mito, that is, negative value higher methylation in young mito; positive value higher methylation in old mito). *n* = 6 mice. Table 9: List of oligonucleotides used in the study.

## Source data

Source Data Fig. 1 Statistical source data.

Source Data Fig. 2 Statistical source data.

Source Data Fig. 3 Statistical source data.

Source Data Fig. 4 Statistical source data.

Source Data Extended Data Fig. 1 Statistical source data.

Source Data Extended Data Fig. 2 Statistical source data.

Source Data Extended Data Fig. 3 Statistical source data.

Source Data Extended Data Fig. 4 Statistical source data.

Source Data Extended Data Fig. 5 Statistical source data.

Source Data Extended Data Fig. 5 Unprocessed scans of gels.

Source Data Extended Data Fig. 6 Statistical source data.

Source Data Extended Data Fig. 7 Statistical source data.

Source Data Extended Data Fig. 8 Statistical source data.

Source Data Extended Data Fig. 9 Statistical source data.

Source Data Extended Data Fig. 10 Statistical source data.

## Data Availability

The data that support the findings of this study are available. Source data are included in the source data files. ISC^mito-O^ and ISC^mito-Y^ RNA-seq and data are available at ArrayExpress under accession no. E-MTAB-13036. ISC^mito-O^ and ISC^mito-Y^ oxidative WGBS-seq data are available at ArrayExpress under accession no. E-MTAB-15119. PC versus ISC mRNA fold change of the DMRs associated with the genes in ISC^mito-O^ was obtained from the RNA-seq data^48^. They are available at ArrayExpress under accession no. E-MTAB-7916. RNA-seq data of Dm-aKG-treated ISCs are available at ArrayExpress under accession no. E-MTAB-15105. E5hmC-seq data are available at ArrayExpress under accession no. E-MTAB-15115. Metabolomics data have been deposited with the MetaboLights^76^ repository with the study identifier MTBLS12349. Source data are provided with this paper.
