# Supplementary material for: Old mitochondria regulate niche renewal via α-ketoglutarate metabolism in stem cells
Source: Nat Metab. 2025 Jul 14;7(7):1344–57. doi: 10.1038/s42255-025-01325-7 (PMC12286850; doi:10.1038/s42255-025-01325-7)
Supplement: Supplementary file 1 — Supplementary Fig. 1. [file 42255_2025_1325_MOESM1_ESM.pdf]

nature metabolism

Supplementary information https://doi.org/10.1038/s42255-025-01325-7

Old mitochondria regulate niche renewal via α-ketoglutarate metabolism in stem cells

In the format provided by the authors and unedited

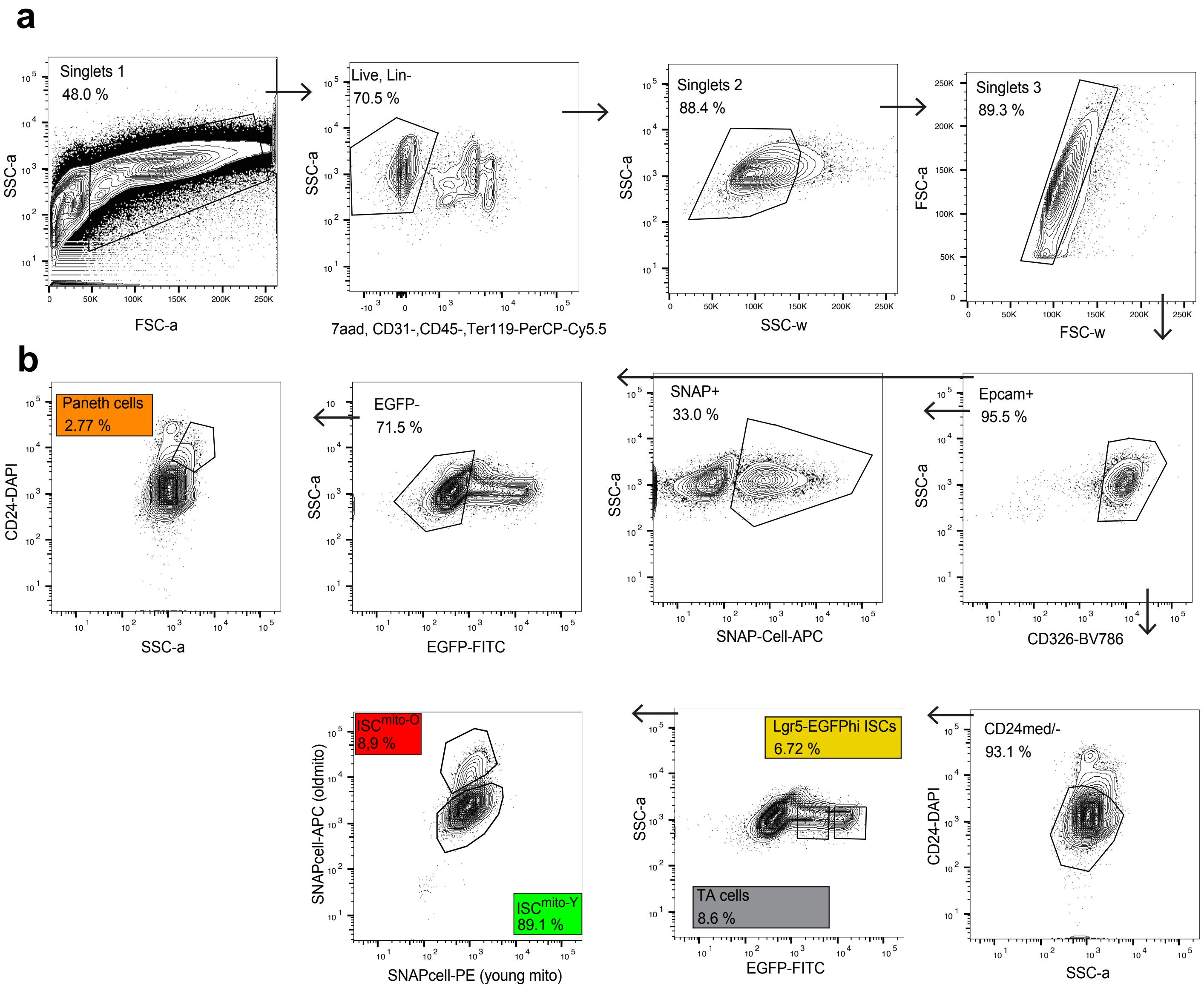

Supplementary figure 1. FACS gating strategies

a, Gating strategy for isolating live single cells. **b,c**, Singlets 3 is the parent gate to all gates. **b**, Gating strategy for SNAP-cell+ epithelial cells and Paneth cells. **c**, Gating strategy for ISCs, ISCs^{mito-O} and ISCs^{mito-Y}. All data is represented as 5% contour plots with outliers except SSCa- EGFP plots and CD24 – SSCa plots and SNAP-cell old – SNAP-cell young, which are presented as 2% contour plots with outliers.
