# Supplementary material for: Old mitochondria regulate niche renewal via α-ketoglutarate metabolism in stem cells
Source: Nat Metab. 2025 Jul 14;7(7):1344–57. doi: 10.1038/s42255-025-01325-7 (PMC12286850; doi:10.1038/s42255-025-01325-7)
Supplement: Supplementary file 2 — Reporting Summary [file 42255_2025_1325_MOESM2_ESM.pdf]

nature portfolio

Corresponding author(s): Pekka Katajisto

Last updated by author(s): May 1, 2025

Reporting Summary

Nature Portfolio wishes to improve the reproducibility of the work that we publish. This form provides structure for consistency and transparency in reporting. For further information on Nature Portfolio policies, see our Editorial Policies and the Editorial Policy Checklist.

Statistics

For all statistical analyses, confirm that the following items are present in the figure legend, table legend, main text, or Methods section.

n/a	Confirmed	
☐	☒	The exact sample size (*n*) for each experimental group/condition, given as a discrete number and unit of measurement
☐	☒	A statement on whether measurements were taken from distinct samples or whether the same sample was measured repeatedly
☐	☒	The statistical test(s) used AND whether they are one- or two-sided<br>*Only common tests should be described solely by name; describe more complex techniques in the Methods section.*
☒	☐	A description of all covariates tested
☐	☒	A description of any assumptions or corrections, such as tests of normality and adjustment for multiple comparisons
☐	☒	A full description of the statistical parameters including central tendency (e.g. means) or other basic estimates (e.g. regression coefficient) AND variation (e.g. standard deviation) or associated estimates of uncertainty (e.g. confidence intervals)
☐	☒	For null hypothesis testing, the test statistic (e.g. F, t, r) with confidence intervals, effect sizes, degrees of freedom and P value noted<br>*Give P values as exact values whenever suitable.*
☒	☐	For Bayesian analysis, information on the choice of priors and Markov chain Monte Carlo settings
☒	☐	For hierarchical and complex designs, identification of the appropriate level for tests and full reporting of outcomes
☐	☒	Estimates of effect sizes (e.g. Cohen's d, Pearson's r), indicating how they were calculated

Our web collection on statistics for biologists contains articles on many of the points above.

Software and code

Policy information about availability of computer code

Data collection	FACSDiva version 8<br>LAS X (5.0)<br>Xcalibur 4.1.31.9
Data analysis	Microsoft Excel 16.68<br>Graphpad Prism 9<br>FACSDiva version 8<br>FlowJo v10<br>Fiji version 2<br>Slideviewer version2<br>TraceFinder 4.1 SP2 software<br>fastqc version 0.11.8<br>Star versions 2.7.3a 2.7.11a<br>RSeOC version 3.0.1<br>UMI-tools version 1.0.1<br>kallisto version 0.46.1<br>tximport version 1.14.0<br>DESeq2 version 1.26.0<br>DESeq2 v1.40.2<br>limma version 3.42.0

CFX Maestro 1.1 version 4.1
bcl2fastq version 2.20
R v4.3.3
methylkit 1.33.1
Metilene v0.2-8
SortMeRNA v4.3.6
HTSeq v2.0.2

For manuscripts utilizing custom algorithms or software that are central to the research but not yet described in published literature, software must be made available to editors and reviewers. We strongly encourage code deposition in a community repository (e.g. GitHub). See the Nature Portfolio guidelines for submitting code & software for further information.

Data

Policy information about availability of data

All manuscripts must include a data availability statement. This statement should provide the following information, where applicable:

- Accession codes, unique identifiers, or web links for publicly available datasets
- A description of any restrictions on data availability
- For clinical datasets or third party data, please ensure that the statement adheres to our policy

The data that support the findings of this study are available. Source data are in included source data files.
ISCmito-O and ISCmito-Y RNA sequencing and data available at ArrayExpress with accession code:E-MTAB-13036.
ISCmito-O and ISCmito-Y Whole-genome oxidative bisulfite sequencing data available at ArrayExpress with accession code:E-MTAB-15119
Paneth cell vs ISCs mRNA fold changed of the DMRs associating with genes in ISCmito-O was obtained from RNA-sequencing data (Pentinmikko et al.) ArrayExpress with accession code:E-MTAB-7916.
Dm-aKG treated ISCs RNA sequencing data available at ArrayExpress with accession code:E-MTAB-15105
Dm-aKG treated ISCs NEBNext® Enzymatic 5hmC-seq (E5hmC-seq) sequencing data available at ArrayExpress with accession code:E-MTAB-15115
The metabolomics data have been deposited to MetaboLights repository with the study identifier MTBLS12349.
Mouse genome assemblies GRCm38.p6 (mm10) and GRCm39 were used to align sequencing data to the genome, publicly available at (https://www.ncbi.nlm.nih.gov/datasets/genome/GCF_000001635.20/) and (https://www.ncbi.nlm.nih.gov/datasets/genome/GCF_000001635.27/), respectivley

Human research participants

Policy information about studies involving human research participants and Sex and Gender in Research.

Reporting on sex and gender	N/A
Population characteristics	N/A
Recruitment	N/A
Ethics oversight	N/A

Note that full information on the approval of the study protocol must also be provided in the manuscript.

Field-specific reporting

Please select the one below that is the best fit for your research. If you are not sure, read the appropriate sections before making your selection.

☒ Life sciences ☐ Behavioural & social sciences ☐ Ecological, evolutionary & environmental sciences

For a reference copy of the document with all sections, see nature.com/documents/nr-reporting-summary-flat.pdf

Life sciences study design

All studies must disclose on these points even when the disclosure is negative.

Sample size	No statistical methods were used to pre-determine sample sizes but our sample sizes are similar to those reported in previous publications(DOI:https://doi.org/10.1038/nature21673 , DOI:https://doi.org:10.1038/nature09637, DOI:https://doi.org:10.1016/j.stem.2017.02.007)
Data exclusions	No data was excluded for other reasons than for being technically inadequate or unusable due to mistakes.
Replication	All experiments were successfully reproduced with biological independent replicates a minimum of three times, with exact replicate number specified in each figure legend

Randomization	All animals were randomly allocated to experimental groups, but without a formal randomization.
Blinding	Data collection and analysis were not performed blind to the conditions of the experiments.

Reporting for specific materials, systems and methods

We require information from authors about some types of materials, experimental systems and methods used in many studies. Here, indicate whether each material, system or method listed is relevant to your study. If you are not sure if a list item applies to your research, read the appropriate section before selecting a response.

Materials & experimental systems

n/a	Involved in the study	
☐	☒	Antibodies
☐	☒	Eukaryotic cell lines
☒	☐	Palaeontology and archaeology
☐	☒	Animals and other organisms
☒	☐	Clinical data
☒	☐	Dual use research of concern

Methods

n/a	Involved in the study	
☒	☐	ChIP-seq
☐	☒	Flow cytometry
☒	☐	MRI-based neuroimaging

Antibodies

Antibodies used

CD31–PerCP-Cy5.5, BD, Mec13.3 1:500
CD45–PerCP-Cy5.5, BD, 30-F11 1:500
Ter119–PerCP-Cy5.5, BD, Ter119 1:500
CD326–BV786, BD, G8.8, 1:500
CD24–BV421, BD, M1/69 1:500
Lysozyme, DAKO, EC3.2.1.17 1:500
E-cadherin BD, 610181 1:500
Chromogranin A, abcam, ab15160 1:500
Mucin 2, Santa-cruz Biotechnology, H-300, sc-15334 1:500
5hmC, Active motif, AB_10013602 1:500
anti-rabbit-Alexa-488, ThermoFischer, A11008
anti-rabbit-Alexa-594, ThermoFischer, A11012
anti-rabbit-Alexa-647, ThermoFischer, A21244
anti-mouse-Alexa-488, ThermoFischer, A28175
anti-mouse-Alexa-594, ThermoFischer, A28175
anti-rat-Alexa-488, Thermofisher, A21470
anti-rat-Alexa-647, Thermofisher, A78947

Validation

All used antibodies were commercially available and thus were validated by the manufacturer, data available on the manufacturer's website, the validation information from the manufacturer for each antibody is linked below.

For flow cytometry antibody panel was designed so as to minimize spectral overlap and single stained samples were run to run apply compensation of less than 2%. These conjugated antibodies have been used previously in the laboratory with similar results. For flow cytometry: CD31–PerCP-Cy5.5, BD, Mec13.3(Manufacturer validation;https://www.bdbiosciences.com/content/dam/bdb/products/global/reagents/flow-cytometry-reagents/research-reagents/single-color-antibodies-ruo/562xxx/5628xx/562861_base/pdf/562861.pdf), CD45–PerCP-Cy5.5, BD, 30-F11(Manufacturer validation;https://www.bdbiosciences.com/content/dam/bdb/products/global/reagents/flow-cytometry-reagents/research-reagents/single-color-antibodies-ruo/550xxx/5509xx/550994_base/pdf/550994.pdf), and Ter119–PerCP-Cy5.5, BD, Ter119(Manufacturer validation;https://www.bdbiosciences.com/content/dam/bdb/products/global/reagents/flow-cytometry-reagents/research-reagents/single-color-antibodies-ruo/560xxx/5605xx/560512_base/pdf/560512.pdf) stain endothelial cells, all hematopoietic linages (excluding platelets and erythrocytes), and erythroid linages respectively. Staining was observed in cells that were mutually exclusive with CD326–BV786, BD, G8.8(Manufacturer validation;https://www.bdbiosciences.com/content/dam/bdb/products/global/reagents/flow-cytometry-reagents/research-reagents/single-color-antibodies-ruo/740xxx/7409xx/740958_base/pdf/740958.pdf) that marks all epithelial cells. For CD326 the opposite conclusion was used and the Lgr5-EGFP transgene signal was observed in CD326. For CD24–BV421, BD, M1/69(Manufacturer validation;https://www.bdbiosciences.com/content/dam/bdb/products/global/reagents/flow-cytometry-reagents/research-reagents/single-color-antibodies-ruo/562xxx/5625xx/562563_base/pdf/562563.pdf), high staining was observed in Paneth cells, phenotypically identified by high side scatter, and were validated by microscopy to contain phenotypical large granules.

For Lysozyme, DAKO, EC3.2.1.17antibody, specific staining was observed in phenotypic Paneth cells (large granular cells at the bottom of crypts of the small intestine)
For E-cadherin BD, 610181(Manufacturer validation;https://www.bdbiosciences.com/content/dam/bdb/products/global/reagents/microscopy-imaging-reagents/immunofluorescence-reagents/610xxx/6101xx/610182_base/pdf/610181.pdf) stained membranes of epithelial cells and has been used in the laboratory before with similar results.

For Chromogranin A, abcam, ab15160(Manufacturer validation;https://doc.abcam.com/datasheets/active/ab15160/en-us/chromogranin-a-antibody-ab15160.pdf) antibody stains a rare (1-2%) hormone secreting cell population with a distinct

morphological characteristic of large basal to luminal ratio surface ratio.
F
or Mucin 2, Santa-cruz Biotechnology, H-300, sc-15334 antibody stains large mucous producing cells and a mucus layer on top of the epithelium along the crypt to villus axis.

For 5hmC, Active motif, AB_10013602(Manufactueres validation:https://www.activemotif.com/documents/tds/39769.pdf)stains the DNA modification 5-hydroxymethylcytosine. Signal increase was seen after treatment with alpha-ketoglutarate a known ten-eleven translocation methylcytosine dioxygenases activator. For secondary antibodies, replicate samples without primary antibodies was used to ascertain primary specificity.

Eukaryotic cell lines

Policy information about cell lines and Sex and Gender in Research

Cell line source(s)	293fT cells we purchased from ThermoFisher, R70007
Authentication	No authentication of the cell line was performed
Mycoplasma contamination	The cell was routinely tested for mycoplasma which was negative
Commonly misidentified lines (See ICLAC register)	N/A

Animals and other research organisms

Policy information about studies involving animals; ARRIVE guidelines recommended for reporting animal research, and Sex and Gender in Research

Laboratory animals	Mouse (Mus musculus):<br>Wild type and Lgr5-EGFP-IRES-CreERT2 and Rosa26(LSL-SNAPtag-omp25) were maintained in C57BL/6J background.<br>Mice were purchased from Innotiv , C57BL/6JRccHsd<br>In all experiments, animals used were between 3 and 25 months of age. Young mice between the ages 3-6 mice were used in all experiments, Ages of the Aged mice are specified in in the relevant figures.
Wild animals	No wild animals were used
Reporting on sex	Both sexes were used throughout the study. Sex was considered during the experimental design to ensure balanced representation in all groups. Experimental replicates are always individual mice and cells are compared to cells originating from the same mouse in a pair-wise fashion, intrinsically producing sex-matched comparisons.
Field-collected samples	No field samples were collected
Ethics oversight	Animal studies were approved by the National Animal Ethics Committee of Finland (ELLA) and conducted with the support of the HiLIFE Laboratory Animal Centre Core Facility, University of Helsinki, Finland, under institutional guidelines. The licence numbers covering all related work are ESAVI-7011-2019 and ESAVI/18179/2020.

Note that full information on the approval of the study protocol must also be provided in the manuscript.

Flow Cytometry

Plots

Confirm that:

☒ The axis labels state the marker and fluorochrome used (e.g. CD4-FITC).

☒ The axis scales are clearly visible. Include numbers along axes only for bottom left plot of group (a 'group' is an analysis of identical markers).

☒ All plots are contour plots with outliers or pseudocolor plots.

☒ A numerical value for number of cells or percentage (with statistics) is provided.

Methodology

Sample preparation	Mouse primary intestinal epithelial cells were isolated by EDTA mediates chelation of magnesium and calcium of minced tissue followed by gentle mechanical dissociation and enzymatic treatment with TrypLE Express enzyme to yield a single cell suspension.
Instrument	FACSAriaIII Fusion (BD) was used to analyze and collect data
Software	FACSDiva v8 was used to collect data and FlowJo v10 was used to analyze cellular frequencies

Cell population abundance	Lgr5+ stem cell populations were ~99 % pure based on fluorescent microscopy of the post-sort fraction. Paneth cells were ~90% pure based on phase contrast microscopy (granulated morphology) and Lysozyme staining of the post-sort fraction. Culture of Paneth cells alone was performed in order to analyze the frequency of Paneth-ISC doublets.
Gating strategy	Single cells were gated by using FSC-A,FSC-W,SSC-A and SSC-W parameters as detailed in Extended data Figure 2. Initially the right population was identified by overlaying Lgr5-EGFP on SSC-A vs FSC-A gate. Similar overlay were used to ensure that doublets were not included in the downstream gating. For fluorescent markers, positive populations were identified by comparing to unstained control sample from the same tissue

☒ Tick this box to confirm that a figure exemplifying the gating strategy is provided in the Supplementary Information.
