# Supplementary material for: Old mitochondria regulate niche renewal via α-ketoglutarate metabolism in stem cells
Source: Nat Metab. 2025 Jul 14;7(7):1344–57. doi: 10.1038/s42255-025-01325-7 (PMC12286850; doi:10.1038/s42255-025-01325-7)
Supplement: Supplementary file 13 — Unprocessed scans of gels. [file 42255_2025_1325_MOESM13_ESM.pdf]

a

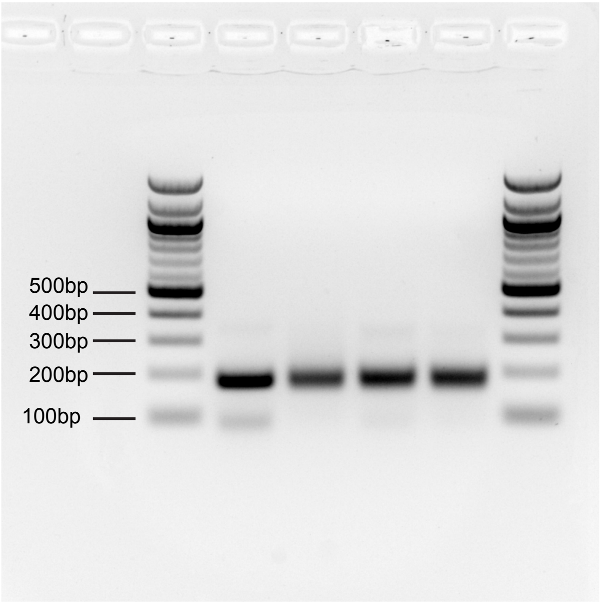

b

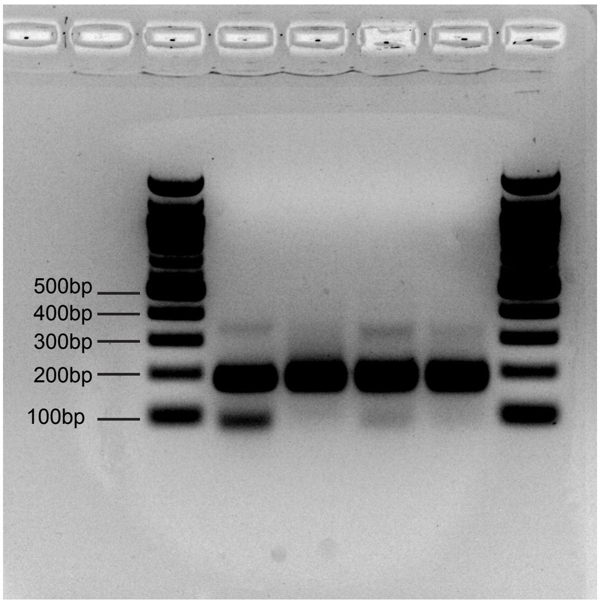

Source Data Extedended Data Fig 5d. Uncropped scans of agarose gel. a, Uncropped Ethidium Bromide stained agarose gel relative to Extended Data Fig. 7d. **b,** Overexposed scan of gel Ethidium Bromide stained agarose gel relative to Extended Data Fig. 7d
